# Structural design and temperature control enabling high sensitivity nanomaterial-based three-electrode gas sensors

**DOI:** 10.1038/s41598-024-76678-2

**Published:** 2025-07-01

**Authors:** Muhammad Waqas, Yong Zhang, Saif Aldeen Saad Obayes Alkadhim, Xiaoyu Li, Liang Xie

**Affiliations:** 1 State Key Laboratory of Electrical Insulation and Power Equipment, Xi’an Jiaotong University, Xi’an, 710049 P. R. China; 2 School of Electrical Engineering, Xi’an Jiaotong University, Xi’an, 710049 P. R. China; 3 School of Instrument Science and Technology, Xi’an Jiaotong University, Xi’an, 710049 P. R. China

**Keywords:** Gas discharge physics, CNTs ionization sensor, Nanomaterial structure optimization, Electrostatic field model, MEMS gas sensor, Electrical and electronic engineering, Electronic devices, Nanosensors, Sensors

## Abstract

Ionization based gas sensors using nanomaterials hold significance in monitoring gases but often suffer from issues such as excessive positive ion bombardment, which reduces lifespan, current collection, and detection accuracy. This study introduces a two-dimensional plasma discharge current model based on particle mass conservation, electron energy conservation, and Poisson equations to evaluate the discharge characteristics and electric fields distribution effects on sensor performance across various morphologies and cathode nanomaterial quantities, with experimental validation. The results indicated that the diffusion aperture diameter structure adjustment in sensor electrode surface maintains a high reverse electric field *E*_1_ around the nanotips of the cathode, accelerated maximum positive ions away from nanomaterial, which reduces positive ion bombardment. The novel Φ = 1.2 × 9 mm diffusion aperture sensor with a 150 nm gold nanostructured cathode effectively directed approximately ~ 2/3 of positive ions from the ionization to the collection region, mitigating corrosion and bombardment effects. Compared to previous structure, this novel sensor shows three times greater sensitivity to H_2_, C_2_H_2_, CH_4_, SO_2_, NO, and O_2_, with enhanced detection ranges down to ppm, ppb, and ppt levels.

**Supplementary Information:**

The online version contains supplementary material available at 10.1038/s41598-024-76678-2.

In 2001, we fabricated a dual-electrode gas sensor based on a carbon nanotube (CNTs)^1^. However, the gas sensor operated in a self-sustaining discharge, causing damage to the nanomaterial^2–10^. To improve the performance, we developed a weakly ionized gas sensor structure in 2013^11^. The primary electrode of this sensor is a nanomaterial. In this configuration, the vertically aligned electrodes operate in a non-self-sustaining discharge. When the sensor is exposed to the gases, the emission of initial electrons from nanotips leads to collisions with gas molecules, generating electrons and positive ions through the *α* process (Fig. 1a). Nonetheless, this configuration demonstrated higher sensitivity to a range of gases (H_2_, C_2_H_2_, CH_4_, SO_2_, NO, O_2_ and C_2_H_4_) and their mixtures^12–16^. The vulnerability of nanomaterial-based sensor configurations to positive ion bombardment poses a significant challenge due to the high field enhancement factor of the nanomaterials. These materials inherently facilitate the efficient convergence of electric fields, leading to self-sustaining electrode discharges (ionization process) at reduced voltage levels^11,16–19^. If the sensor’s structure fails to effectively collect maximum positive ions^11–16^, as illustrated in Fig. 1a, these ions enter a self-sustaining condition and extensively bombard the cathode material. This phenomenon significantly reduces the operational longevity of the sensor, ultimately compromising its performance and limiting its practicality in industrial applications^20,21^.

**Figure 1:**
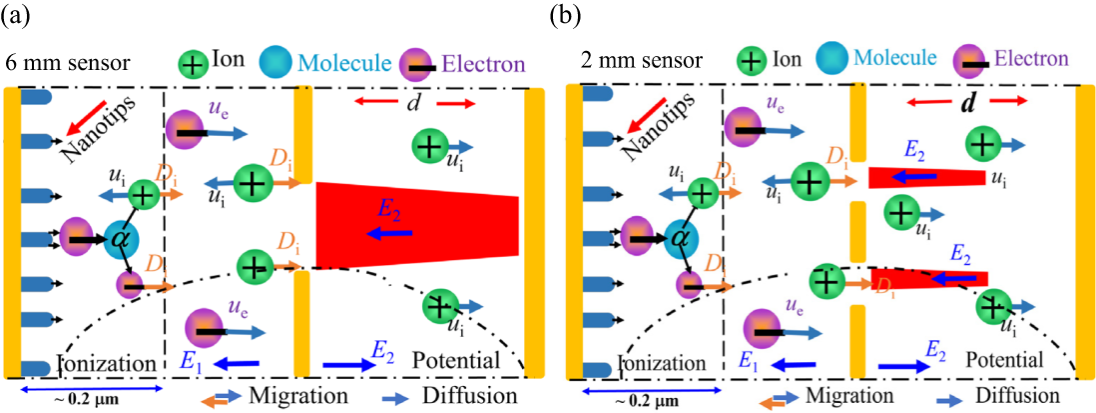
Influence of the electrode structure on electric field distribution. (**a**) Schematic of Φ = 6 mm sensor structure, (**b**) schematic of Φ = 2 mm structure.

Recent concerns about sensor performance mainly address material sensitivity, but there is a notable lack of focus on optimizing nanomaterial-based sensor structures. Studies have either analyzed electric field distribution in sensor designs or investigated gas discharge mechanisms in various electrode configurations. However, there is a significant oversight in addressing the design and optimization of nanomaterial-based sensor structures^11–14^. Some studies have concentrated on analyzing electric field distribution in sensor structures^22–24^, others have delved into the gas discharge mechanism in cylindrical and bare parallel electrodes^17,19,25–31^. This divide highlights the need for a more integrated approach that considers both material performance and structural design to advance the field. Moreover, the unique nano-scale structure of nanomaterials within the film leads to the generation of highly nonlinear electric fields near the cathode nanotips^11,14,17,19^, complicating the physics involved. Additionally, ionized gas sensors utilizing nanomaterials operate via direct current discharge under atmospheric pressure, triggering numerous chemical reactions and species^11,14,17,19,24,32,33^. Consequently, establishing a model to comprehensively explore the gas discharge mechanism poses significant challenges within an individual sensor structure. As a result, this phenomenon remains not fully understood, contributing to sensors with shorter lifespans and lower collecting currents.

Here, we propose a comprehensive approach to optimize the nanomaterial-based gas sensor performance by developing a two-dimensional plasma discharge current model based on particle mass conservation, electron energy conservation, and Poisson’s equations. This model explores how structural changes affect reverse (*E*_1_) and forward (*E*_2_) electric field distributions across the electrode surface. Our simulations reveal that the diffusion aperture diameter significantly influences electric field distribution. Additionally, we assess the discharge characteristics of graphene, carbon nanotubes (CNTs), and 150 nm gold nanomaterials, focusing on their ionization efficiency and corrosion resistance. Our analysis highlights the importance of the cathode nanotips’ structure in enhancing the electric field and examines how different power voltages and temperature impact sensor performance. Validated through experiments, our novel three-electrode sensor shows superior performance compared to previous designs across various gases (H_2_, C_2_H_2_, CH_4_, SO_2_, NO, and O_2_) and conditions. This method systematically advances the design of nanomaterial-based gas sensors, improving both sensitivity and longevity.

## Materials, simulation, and experimental procedure

The optimization of the gas sensor is achieved through the integration of simulation and experimental methods, applied to two distinct CNT-based sensor structures. This section details the fabrication and development of these sensors, including the operation of a two-dimensional plasma discharge current model. It also covers the structural composition, performance impacts, and underlying operational principles of the sensors.

### Fabrication of the sensors

To evaluate how structural composition affects gas sensor performance, we fabricated two sensor structures from a 450 μm thick N-type nano-crystalline silicon wafers using masking, photolithography, and etching process or MEMS process (Supplementary Fig. s1A)^11,12^. Both sensors have electrodes that are 27 mm long and 8 mm wide but differ in the number of holes, as shown in Fig. 1a and b, with diffusion apertures of Φ = 6 mm and Φ2 × 6 mm for the extracting electrode. Vertically aligned electrodes were coated with CNTs, grown by thermal chemical vapor deposition (TCVD), with diameters of 50 nm and 150 nm and lengths of approximately 5 μm. A 50 nm Ti film, a 400 nm Ni film, and a 125 nm Au film were deposited on the electrodes via magnetron sputtering. These films were then rapidly annealed in a low vacuum (~ 3 Pa) at 450 °C for 50 s to isolate the electrodes and enhance discharge current^11,14^. Electrode separations (*d*) were adjusted using polyester insulation strips, and the sensors were tested to assess the impact of structural variations on performance.

### Principle of operation

The sensor structure facilitates a non-sustained gas discharge near the cathode nanotips in response to the applied fields. Due to *U*_c_ < *U*_e_, two opposing electric fields (*E*_1_ and *E*_2_) are generated between the upper and lower gaps of electrodes (see supplementary Fig. s2). The reverse electric field (*E*_1_), reaching approximately (10 ^ 7 V/m) within 0.2 μm of the nanotips, promotes substantial electron emission from the carbon nanotubes. These electrons ionized with gas molecules under *E*_1_, producing a large number of positive ions. These ions, after collision and diffusion, overcome the weak field near the extracting electrode and move toward it with diffusion coefficient *D*_i_. They are then accelerated by forward electric field *E*_2_ toward the collecting electrode, generating the collecting current *I*_c_, which is measured using the NI PXI-4071 digital multimeter. Consequently, the frequency of ion bombardment on the nanomaterials is significantly reduced. *I*_c_ ​represents approximately ~ 1/3 of the total current *I*, which is linearly correlated with ionization coefficient *α*^11,15^.

1 $$\alpha =AP{e^{ - BP/E}}$$

Here, *A* and *B* are constants dependent on gas composition and temperature. As the electric field, *E* approaches a critical value, *α* increase with gas pressure *P*^11^. Concurrently, the collecting current *I*_c_, which is a fraction of the total discharge current *I*, also rises with *α* in non-self-sustaining discharges.

2 $$I={I_0}{{\text{e}}^{\alpha d}}$$

Where *I*_0_ is the initial discharge current, *d* is electrode spacing, when the ionization sensor exposes to the target gases, which are mixed with N_2_. Notably, the gases such as H_2_, C_2_H_2_, SO_2_, CH_4_, CO, O_2_ and NO, featuring an initial ionization coefficient higher than 10.3 eV (Table 1)^11^, are highly resistive to ionize compared to N_2_. However, the ionization process predominantly relies on the ionization of two meta-stable states of N_2_ at 6.8 eV of N_2_(A^3^∑_u_^+^), and 8.4 eV of N_2_(a′^1^∑_u_^-^) respectively. When the targeted gases are present in the N_2_, they quench and revert the meta-stable states of N_2_to the ground state^11–16^, which inhibits the ionization of N_2_. Consequently, the collecting current (*I*_c_) decreases proportionally with increasing target gas concentration.

**Table 1:** Ionization energies of gasses

Gas	*N*_2_	CO	CH_4_	C_2_H_2_	H_2_	O_2_	SO_2_	NO	*N*_2_(A^3^∑_u_^+^)	*N*_2_(a′^1^∑_u_^−^)
***ε***_**n**_**/eV**	15.5	14.01	12.65	11.40	15.43	12.2	12.35	9.26	6.2	8.4

### Development of three-electrode ionization sensor fluid model

**Figure 2:**
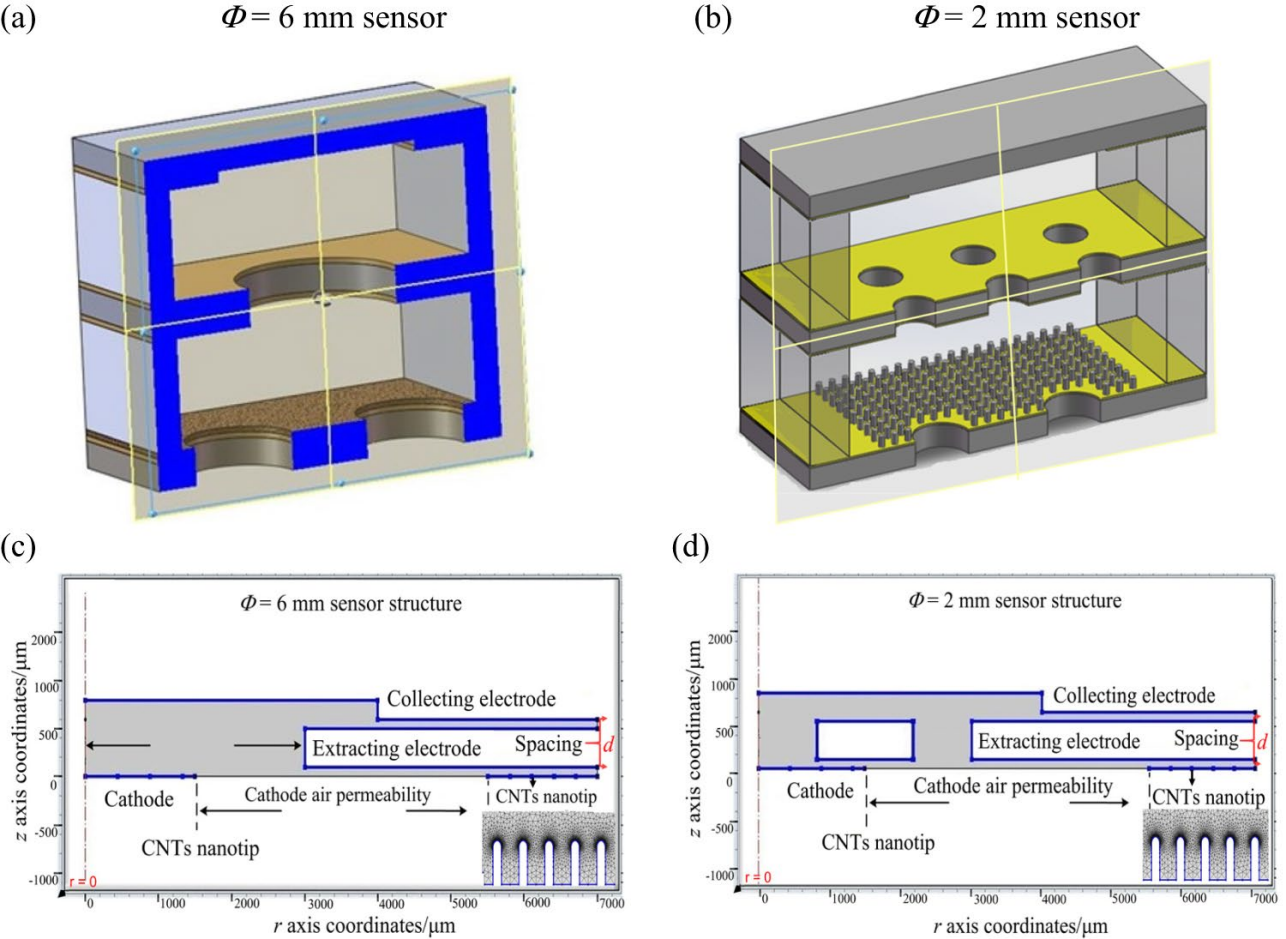
Simulation models of sensor, (**a**) Schematic diagram of the longitudinal cross section of the three-dimensional diagram of the Φ = 6 mm sensor, (**b**) Φ = 2 mm sensor. (**c**) The two-dimensional asymmetric simulation model of Φ = 6 mm sensor. (**d**) The two-dimensional asymmetric simulation model of Φ = 2 mm sensor structure.

To evaluate the impact of distant electrodes structure on sensor performance, we developed simulation models with different electrode configurations (see Fig. 2). These models included sensors with identical electrode dimensions but varying hole numbers, as shown in Fig. 2a and b, featuring extracting electrodes with diffusion apertures of Φ = 6 mm and Φ = 2×6 mm, respectively. Asymmetric models for these configurations were created using COMSOL’s electrostatic (*E*_S_) and plasma discharge (DC) modules (see Fig. 2c, and d). Boundary conditions were set according to the electrostatic field Eq. (3), where we used the Laplace Eq. (4) to calculate electric field distributions both (*E*_1_ and *E*_2_) electric fields.

3 $${\mathbf{E}}= - \nabla V$$

**Figure 3:**
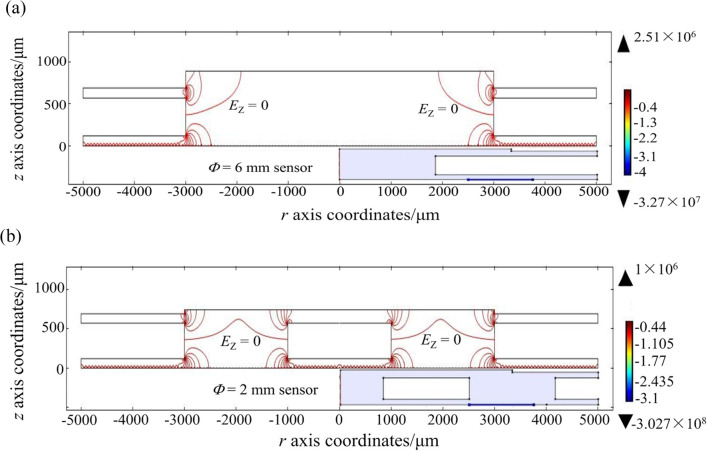
Simulation result of electrostatic field distribution between the electrodes of (**a**) Φ = 6 mm sensor and (**b**) Φ = 2 mm sensor in width direction.

Here, ***E*** represents the electric field strength, and ***V*** denotes the voltage. To analyze the sensor’s performance, we employed three key equations: the mass-conservation Eq. (4), the electron energy conservation Eq. (5), and the Poisson Eq. (6). These equations allow for the calculation of discharge parameters such as electron density *n*_e_, positive ion density *n*_+_, cathode current density, maximum forward and reverse electric field strength and collecting current density *j*_c_ (see Fig. 3 and Table 2).

4 $${{\mathbf{\Gamma }}_{\text{i}}}=\operatorname{sgn} \left( {{q_{\text{i}}}} \right){n_{\text{i}}}{\mu _{\text{i}}}{\mathbf{E}} - {D_{\text{i}}}\nabla {n_{\text{i}}}$$

5 $$\frac{{\partial {n_{{\varepsilon}}}}}{{\partial t}}+\nabla {{\mathbf{\Gamma }}_{{{\varepsilon}}}}=-{\mathbf{E}} \cdot {{\mathbf{\Gamma }}_{\text{e}}}-\sum\limits_{{\text{j}}} {\Delta {E_{\text{j}}}{R_{\text{j}}}} - \sum\limits_{{\text{k}}} {\Delta {E_{\text{k}}}{R_{\text{k}}}}$$

6 $${\varepsilon _{\text{0}}}{\nabla ^2}V= - \sum\limits_{{\text{i}}} {{q_{\text{i}}}{n_{\text{i}}}}$$

**Table 2:** Electrostatic field simulation results

The sensor structure	Conditions	*E*_1max_ (V/m)	*E*_2max_ (V/m)	*n*_emax_ (1/m^3^)	*n*_+max_ (1/m^3^)	*j*_c_ (A/m^2^)	*j*_cathode_ (A/m^2^)
6 mm	Width Direction	1.27 × 10^8^	1.53 × 10^7^	1.20 × 10^11^	1.01 × 10^14^	1.0 × 10^−4^	1.81 × 10^15^
2 mm		4.03 × 10^7^	6.46 × 10^5^	1.49 × 10^11^	1.03 × 10^14^	5.01 × 10^−4^	3.24 × 10^14^
6 mm	length Direction	1.37 × 10^8^	1.58 × 10^7^	1.27 × 10^11^	1.06 × 10^14^	1.2 × 10^−4^	3.05 × 10^15^
2 mm		4.20 × 10^7^	6.55 × 10^5^	1.62 × 10^11^	1.09 × 10^14^	6.39 × 10^−5^	4.61 × 10^14^

Where *Γ*_ε_ electron energy flux, *n*_ε_ is the electron energy density, *S*_ε_ is the energy loss or gain due to collisions, *E* is the electric field, *ε*_0_ is vacuum permittivity, *e* is the elementary charge, *n*_i_ is the mass density *i* (N_2_^+^, N_4_^+^, N_2_(A^3^∑_u_^+^), N_2_(a′^1^∑_u_^−^) of species in nitrogen background, *D*_i_ is the diffusion coefficient and *µ*_i_ is the mobility coefficient (movement of charge particle define as Fig. 1a and b), Δ*E*_j_ and *R*_j_ are the electron energy loss and corresponding reaction rate of inelastic collision *j*, respectively, Δ*E*_k_ and *R*_k_ are the electron energy loss and corresponding reaction rate of elastic collision *K*, *j*, *q* is the charge of species, *U* is the potential and *N* is the gas density. We incorporated N_2_ and other gases fundamental particles and chemical reactions in the simulation (Table S1). Through solving the equations, we were able to calculate the maximum fields, spatial distributions of electrons and positive ions (*n*_+_), cathode current density and the collecting current density (*j*_c_). Results in Fig. 3, s2, s3 and Table 2 provide the insights into discharge dynamics, showing how changes in electrode structure impact ionization efficiency and overall sensor performance.

## Results and discussion

### Simulation analysis and their implication

Two-dimensional schematic and simulation models for distant structures are depicted in Fig. 2. Under the conditions of *U*_e_ = 120 V, *U*_c_ = 10 V, and *d* = 120 μm, the analysis revealed a uniform longitudinal electric field, *E*_z_, along each line *E*_z_ = 0, which acts as a demarcation between two regions. Above this line (where *E*_z_ > 0), the reverse electric field (*E*_1_) promotes the movement of positive ions towards the collecting electrode. Conversely, below this line (where *E*_z_ < 0), the positive electric field (*E*_2_) impedes this movement, as illustrated in Fig. 3, Supplementary Fig. S3 and Table 2, for the width direction and the length direction electric field distribution. By applying these longitudinal electric field assumptions, we investigated the impact of electrode structure on sensor performance. The results of this investigation are presented in Table 2 and have been experimentally validated.

To validate the fluid model, we applied boundary conditions to a 6 mm sensor and used ionization energies of gases (Table 1) to calculate collector current densities for CO and CH_4_ at different concentrations, temperatures, and supply voltages, as shown in Table 4. The experimental and simulation results for both gases showed consistent trends across different conditions, confirming the fluid model’s accuracy in measuring gas concentrations.

### Effects of electrodes structure on sensor performance

The two-dimensional electrostatic field simulations result for the sensors are illustrated in Fig. 3 and S2, reveal distinct patterns in the width and length directions. For the 6 mm sensor (Fig. 3a), the *E*_1_ is absent at the center of the extraction hole and at the center of the collecting electrode. In contrast, the 2 mm sensor structure exhibits a small central region without *E*_1_ on the extracting electrode, but *E*_1_ is present across the entire collecting electrode (Fig. 3b). These findings are supported by the length-direction simulation results in Fig. S2B and summarized in Table 2. Additionally, the 2 mm sensor has an average current density approximately 3.3 times higher than that of the 6 mm sensor (see Fig. S3). Furthermore, the cation density at the cathode carbon tube in line 2 is significantly lower than in line 1 (see Fig. S4), indicating that a higher reverse electric field strength correlates with increased collecting and cathode current densities.

**Figure 4:**
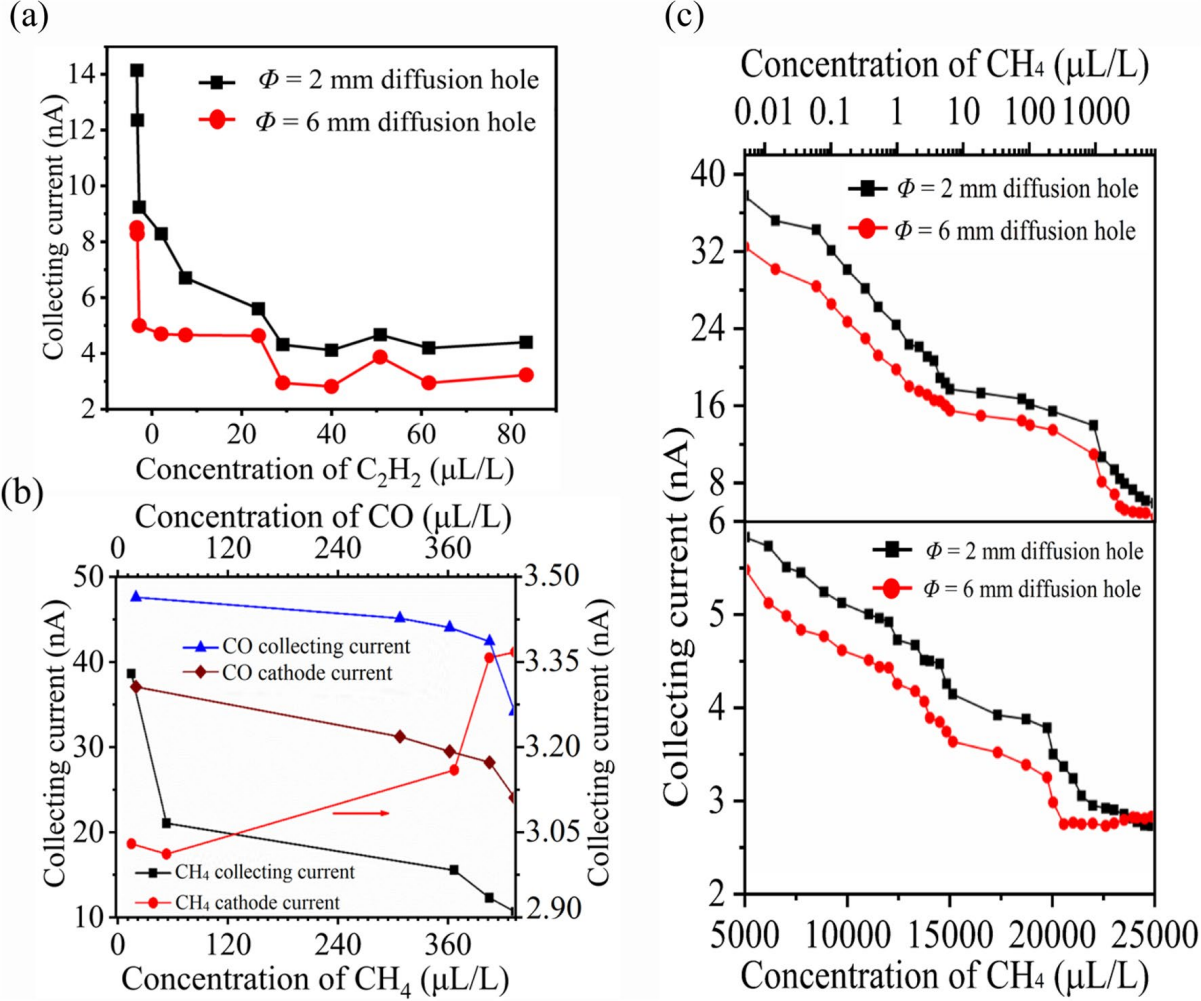
Experimental verification of influence of electrode structure. (**a**) In case of *Φ = *2 mm sensor, the positive ionic current decreases in a single-value with increasing C_2_H_2_ concentration at *d* = 120 μm, 90 V *U*_e_, 10 V *U*_c_ and 30°C, In contrasts with the multi-value decrease in ionic current under the same conditions for the *Φ = *6 mm sensor (red). (**b**) The comparison of the cathodic and positive ionic current collection for the *Φ = *2 mm sensor. At 95 μm electrode spacing, 50°C, and 80 V *U*_e_. With the increase of CO and CH_4_ concentration, the average single values of collected current and cathodic current decrease however, the positive ionic collected current is approximately 1.1 ~ 1.5 times higher than the cathodic current for both gases. (**c**) The sensitivity characteristics comparison the of *Φ = *6 mm and *Φ = *2 mm sensors having CNTs with 150 nm thick gold film cathodes. At 75 μm electrode spacing, 30°C, and 120 V *U*_e_, the *Φ = *6 mm sensor exhibits multi-value sensitivity to CH_4_ gas (red), while the collected current decreases in a single value with the increase of CH_4_ concentration (black) for the *Φ = *2 mm sensor.

To validate the simulation results, we tested the gas ionization properties of three-electrode structures. We measured the current (*I*_c_) for C_2_H_2_ gas flowing through Φ = 6 mm and Φ = 2 mm diffusion aperture sensors, using carbon nanotubes (CNTs) as the cathode material. At 30 °C, 90 V of *U*_e_, 10 V of *U*_c_, and d of 120 μm, the Ic of the Φ = 2 mm sensor declined from 15.02 nA to 5.9 nA as C_2_H_2_ concentration increased from 0 to 80 ppm (Fig. 4a). Conversely, the Φ = 6 mm sensor showed a slightly lower current, consistent with our simulation results (Fig. 1b). The reverse electric field (*E*_1_) in the Φ = 2 mm sensor significantly accelerated positive ions, resulting in a higher collecting current.

The ionization sensor selectively extracts approximately ~ 1/3 of positive ions, enabling operation in non-self-sustained discharges^11^. Experiments with CO and CH_4_ gases evaluated cathode current and positive ion collection efficacy in our optimized sensor structure. Under conditions of 50 °C, 75 V *U*_e_, 10 V *U*_c_, and 100 μm spacing, we observed an inverse relationship between increasing CO concentration (Fig. 4b) and reductions in collecting and cathode currents. At 0 µL/L CO in N_2_, *I*_c_ was 49.36 nA and *I*_cathode_ was 43.67 nA. The collected current consistently exceeded the cathode current by a factor of ~ 1.1 to 1.5. Under conditions of 40 °C, 90 V *U*_e_, 10 V *U*_c_, and 120 μm spacing, the CH_4_ sensor showed similar behavior. At 0 µL/L CH_4_, *I*_c_was 60.33 nA and cathode current 7.814 nA, revealing a significant disparity. The 2 mm sensor’s collected positive ion current was ~ 1/2 ~ 2/3 times higher than the 6 mm sensor’s total discharge current^11,14^, aligning with our results in Fig. S3B (cathode current density). A sensitivity test evaluated two sensor structures detecting trace CH_4_ concentrations using gold-coated CNTs cathodes. At 30 °C, 120 V *U*_e_, and 75 μm spacing, the Φ = 2 mm sensor is collecting current decreased from 42.90 nA to 3.90 nA as CH_4_ concentration increased from 0.01 µL/L to 20,000 µL/L (Fig. 4c). The Φ = 6 mm sensor showed consistently lower current with a multi-value decrease trend. These findings validate the sensing mechanisms, demonstrating that smaller aperture size enhances the reverse electric field, improving positive ion collection and reducing ion bombardment in the Φ = 2 mm sensor.

Reduced aperture diameter enhances sensor performance. We developed eight distinct structures (Supplementary Fig. S5) and measured their collecting current densities and ionization characterization under conditions of *d* = 120 μm, *U*_e_ = 120 V, and *U*_c_ = 10 V in pure N_2_. The Φ = 1.2 × 9 mm configuration consistently showed higher collecting current density (*j*_c_) at 1.10 × 10^−3^ A∙m^−2^ compared to Φ = 6 mm and Φ = 2 × 6 mm structures (Table 3). Simulations and experiments confirm that the small diffusion aperture (sensor 7) (Φ = 1.2 mm sensor, Fig. S1B) enhances positive ion collection, improving sensor performance.

**Table 3:** Electrostatic field simulation results

Sensor	Structure changes	*n*_emax_ (1/m^3^)	*n*_+max_ (1/m^3^)	*j*_c_ (A/m^2^)	*I*_c_(nA)
**Collecting electrode groove structure**					
1	200 μm groove	4.88 × 10^11^	9.30 × 10^13^	6.50 × 10^−4^	3.97 × 10^−4^
2	Bare structure	3.32 × 10^11^	4.85 × 10^13^	4.09 × 10^−4^	2.61 × 10^−4^
3	50 μm groove	4.36 × 10^11^	6.10 × 10^13^	4.67 × 10^−4^	2.94 × 10^−4^
4	12 × 12 mm groove	4.61 × 10^11^	6.29 × 10^13^	4.96 × 10^−4^	3.37 × 10^−4^
**Extracting electrode extraction hole structure**					
1	*Φ* = 6 mm	4.88 × 10^11^	9.30 × 10^13^	6.50 × 10^−4^	3.97 × 10^−4^
5	*Φ = *6 × 2 mm	6.87 × 10^11^	1.12 × 10^14^	7.77 × 10^−4^	3.28 × 10^−4^
6	*Φ = *9 × 1.2 mm	2.15 × 10^12^	3.25 × 10^14^	9.99 × 10^−4^	9.22 × 10^−4^
**Cathode diffusion hole structure**					
6	*Φ = *4 × 2 mm	2.15 × 10^12^	3.25 × 10^14^	9.99 × 10^−4^	9.22 × 10^−4^
7	*Φ = *3 × 2 mm	2.28 × 10^12^	3.67 × 10^14^	1.00 × 10^−3^	1.60 × 10^−3^
8	*Φ = *2 × 2 mm	1.99 × 10^12^	3.09 × 10^14^	9.93 × 10^−4^	1.15 × 10^−3^

### Effects of discharge characteristics of cathode material on sensor performance

**Figure 5:**
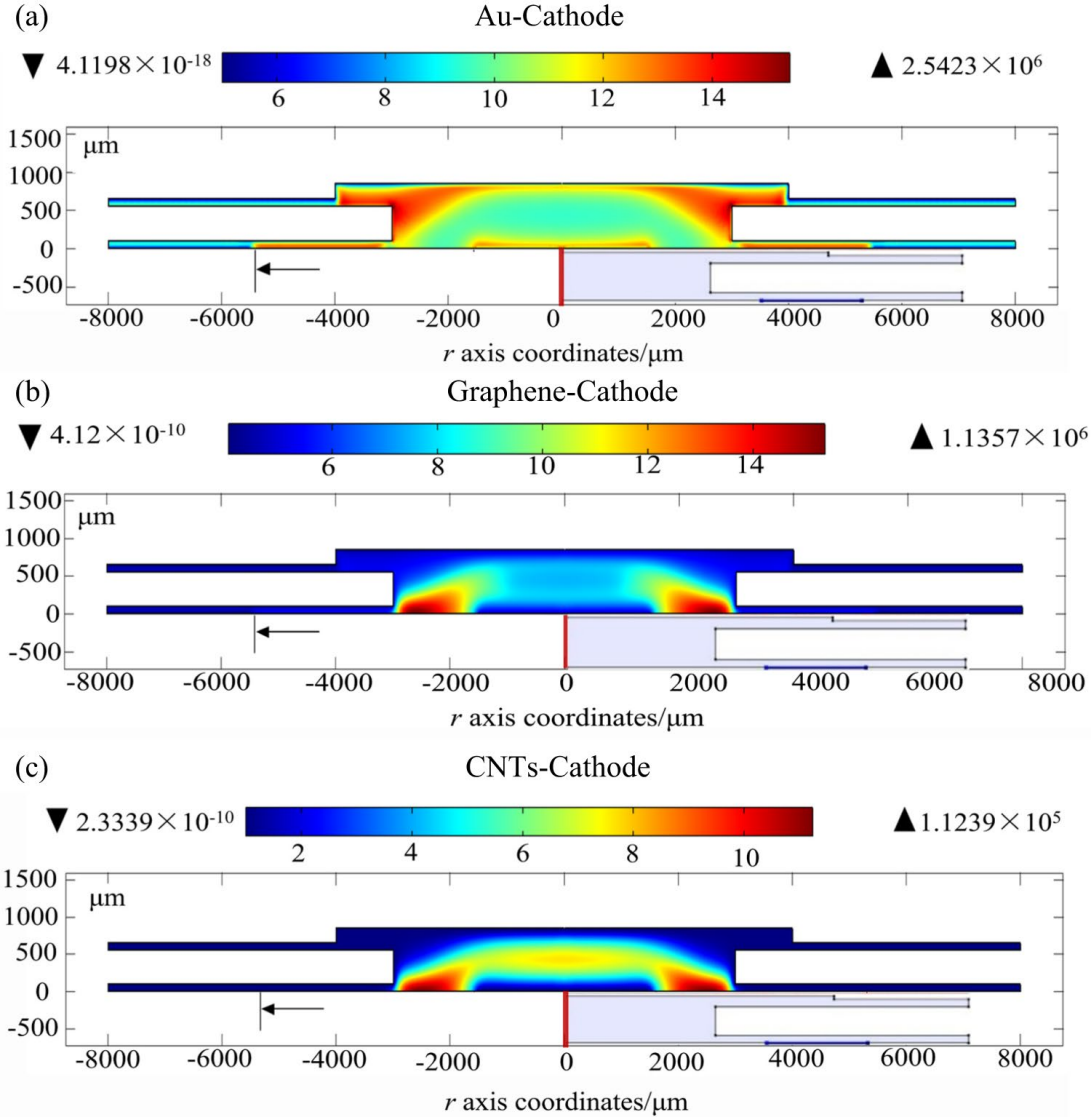
Simulation results on gas discharge characteristics in different cathode-materials (**a**) electron density distribution of Au-cathode sensor, (**b**) electron density distribution of graphene-cathode sensor and **(c)** electron density distribution of CNTs-cathode sensor.

**Figure 6:**
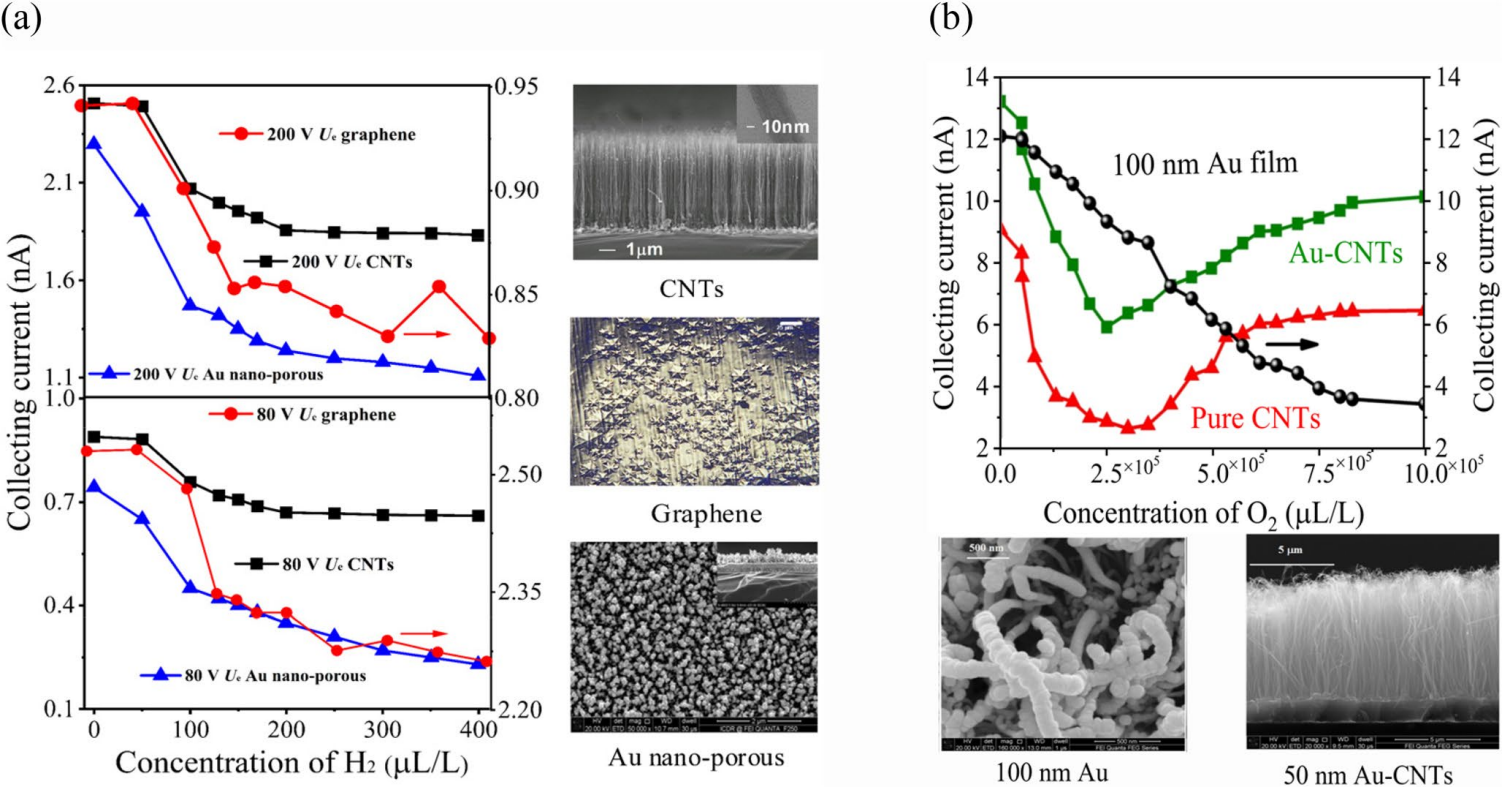
The effect of graphene, gold-coated CNTs, 150 nm gold nanostructure film, and pure CNTs cathode on sensor performance.** (a)** The sensitivity comparison of a graphene, CNTs cathode, and gold nano porous cathode using a *Φ = *6 mm sensor at varying voltages 200 V *U*_e_ and 80 V *U*_e_. At 200 V *U*_e_, 10 V *U*_c_, and 120 μm spacing (*d*), unlike pure CNTs and graphene cathode (which decreased in a multivalve manner), the collecting current of the sensor with Au nanoporous cathode decreased in a linear trend as the H_2_ concentration increased from 0 to 400 ppm. Similar results were observed when the supply voltage was changed to 80 V *U*_e_. **(b)** The performance impact of a *Φ* = 6 mm sensor using different diameter coating of gold nanomaterial and CNTs cathode. At 25°C, 150 V *U*_e_, 1 V *U*_c_, and 100 μm spacing (*d*), the sensor with 150 nm Au-nanomaterial film cathode showed a constant decrease in collecting current as the O_2_ concentration increased from 1 ppm to 10 × 10^5^ ppm, while pure CNTs and Au-CNTs with 50 nm coating cathode sensors exhibited multi-value sensitivity.

Cathode materials in ionization gas sensors inherently facilitate the efficient convergence of electric fields, but are heavily bombarded with positive ions, leading to material damage and reduced sensor performance and lifespan^11–17^. To address this, we studied the sensor’s discharge characteristics using a fluid model (as shown in Fig. 2c and d). Simulations were conducted with the conditions (*U*_e_ = 120 V, *U*_c_ = 10 V, d = 120 μm), and chemical reactions detailed in Table SI. We calculated electron density (*n*_e_), positive ion density (*n*_+_), collecting current density (*j*_c_), and cathode current density using the Laplace equation with gold (Au), graphene, and CNTs as cathode materials. Results (Fig. 5 and Table S2) showed that the ionization properties of Au-cathode sensor remained higher compared to other materials in both structures. To authenticate these findings, we tested sensors with Au-nonporous, CNTs, and graphene cathodes (the sensor fabrication process shown in Fig. S1)^11,14,34^ using a Φ = 6 mm structure (Fig. 6a). At 40 °C, with *U*_e_ = 80 V, *U*_c_ = 10 V, and *d* = 120 μm, the Au cathode sensor showed a single-value decrease in collecting current as H_2_ concentration increased from 1 ppm to 400 ppm, with a sensitivity of -4 pA/ppm. In contrast, CNTs and graphene exhibited multi-value decreases with sensitivities of -2.1 pA/ppm and − 1.8 pA/ppm, respectively. At 50 °C, with *U*_e_ = 200 V, the Au sensor’s sensitivity rose to -9.6 pA/ppm, surpassing CNTs at -5.5 pA/ppm.

Similarly, for the Φ = 2 mm structure, we tested 150 nm Au-coated, 50 nm Au-coated CNTs, and pure CNTs cathodes. At 40 °C, with *U*_e_ = 75 V, the 150 nm Au cathode sensor’s current decreased from 12 nA to 4.6 nA as O_2_ concentration increased to 10 × 10^5^ ppm (Fig. 6b), showing higher sensitivity of -11.09 pA/ppm. CNTs and 50 nm Au-CNTs showed multi-value decreases. The 150 nm Au cathode maintained a higher current, enhancing performance. These results confirm above simulation results that the Au cathode exhibits superior discharge characteristics, increasing charge induction and collecting current density in both senor structure, as confirmed from simulation results.

Furthermore, we also measured the oxidation resistance of cathode material using a Φ = 1.2 mm sensor with CNTs, 150 nm Au, and graphene cathodes under 75 °C, 250 V *U*_e_, 10 V *U*_c_, and 100 μm spacing with continuous discharge concentration of O_2_. The graphene-cathode sensor’s discharge current dropped sharply as O_2_ concentration rose from 0 to 30%, weakening at 30% as illustrated in Fig. S6. The pure CNTs cathode declined at 33% O_2_, losing functionality. In contrast, the 150 nm Au-cathode sensor showed a consistent decrease in discharge current, even at 100% O_2_, demonstrating exceptional stability. This indicates that the 150 nm gold nanomaterial is highly reliable for stability and longevity in ionization-based gas sensors.

### Effects of field emission properties on three-electrode sensor performance

The performance of a sensor is influenced by the height (*h*) and spacing (*x*) of its cathode nanomaterials^19,33^. The electric field intensity near cathode nanotips affects the collecting current (*I*_*c*_), emission current, and gas discharge. For optimizing the Φ = 1.2 mm sensor, we calculated the field enhancement factor for a 150 nm Au-nanostructured cathode. By determining the effective area (*S*_AuF_) with a set radius (*r*) and average spacing (*x*) of Au nanostructures (Fig. S7), and evaluating the electric field intensity. The results suggested in Fig. S6, S7B, when spacing exceeds twice the height, it no longer affects γ. Therefore, the aspect ratio and height were considered to influence the field enhancement factor. Therefore, we fabricated a 150 nm Au nanostructured cathode using TCVD, with a radius of 25 nm, spacing of 300 nm, and height of 600 nm (Fig. S8). This configuration provided strong field strengths, enhancing gas ionization and cathode electron emission. The optimized Φ = 1.2 mm sensor was then tested for emission current under various voltage and temperature conditions.

**Figure 7:**
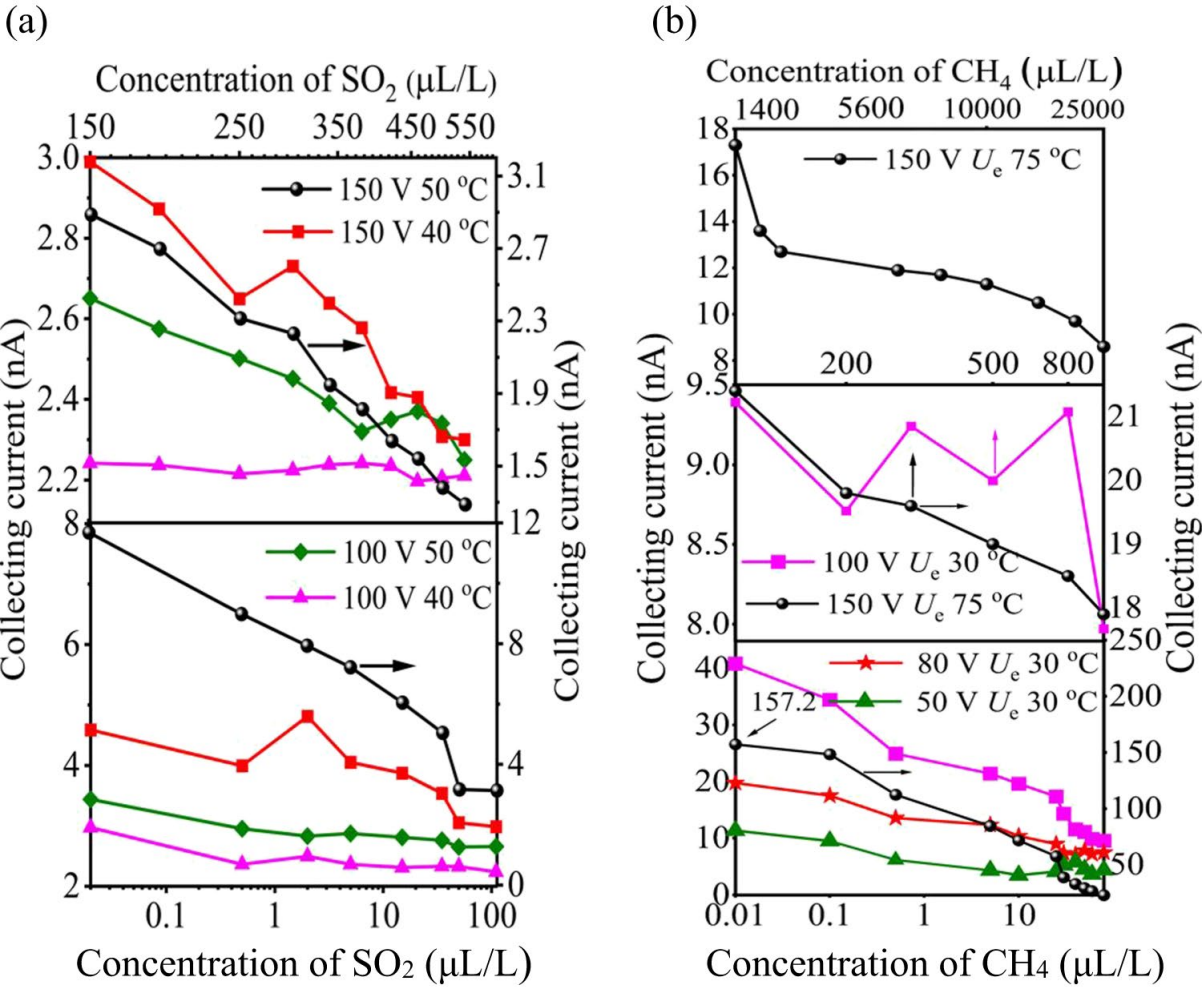
The impact of temperature and electric field on the novel sensor performance. **(a)** The sensor shows single value decrease collecting current to high voltages of 150 V *U*_e_ and temperatures 50°C in SF_6_ mixtures, however it exhibited multi-value collecting current at lower voltages and temperature. **(b)** The collecting current of the CH_4_ sensor variably decreases with increasing concentrations from 0.01 to 25,000 ppm, specifically at 150 V *U*_e_ and 75°C of temperature exhibited higher sensitivity.

Nitrogen gas sensitivity varied with different voltages and temperatures (see Supplementary Table S3, S4). We verified our computational findings by detecting SO_2_ gas at the ppb level in an SF_6_ background, with temperatures of 40 °C to 50 °C and voltages of 100 V and 150 V of *U*_e_. The SO_2_ sensor showed higher sensitivity at 50 °C and 150 V *U*_e_ (Fig. 7a). At 40 °C, the sensor’s *I*_c_ decreased in a multi-valued manner, indicating sensitivity to voltage and temperature changes. Maximum sensitivity was 203 pA/ppm at 0.02 ppm, 150 V *U*_e_, and 50 °C. As SO_2_ concentrations increased from 0.02 to 650 µL/L, the *I*_c_ decreased from 11.92 nA to 1.36 nA at 50 °C and from 4.828 nA to 1.76 nA at 40 °C. We also detected methane in nitrogen to verify emission current changes with temperatures of 30 °C to 75 °C and voltages of 50 V and 150 V.

As illustrated in Fig. 7b, the Φ = 1.2 mm sensor demonstrated high sensitivity to trace methane concentrations, with consistent current decreases from 0.02 to 80 ppm and maximum sensitivity of -31.8 pA/ppm at 0.01 ppm CH_4_, 150 V *U*_e_, and 75 °C. As CH4 concentration increased from 80 to 800 ppm, the current dropped from 22.78 nA to 17.80 nA at 150 V *U*_e_ and 75 °C, but showed multi-valued sensitivity at 100 V *U*_e_ and 30 °C. This behavior aligns with the field-assisted thermal emission law. Therefore, temperature affects the emission current, as pure N_2_has a linear relation^11–16^ with collecting current density *j*_c_*/*A∙m^*−*2^ as confirmed by simulations.

### Sensor performance using two and three gases mixtures

The sensor’s non-linear response between *I*_c_ and *d*enables simultaneous detection of multiple targets^11,14^. We employed two and three novel 1.2 mm sensors with varying electrode separations to measure CH_4_-CO and H_2_-C_2_H_2_ mixtures in N_2_. We also demonstrated how different power supplies affect the sensor’s ability to detect three-gas mixtures at ppm and ppt levels.

**Figure 8:**
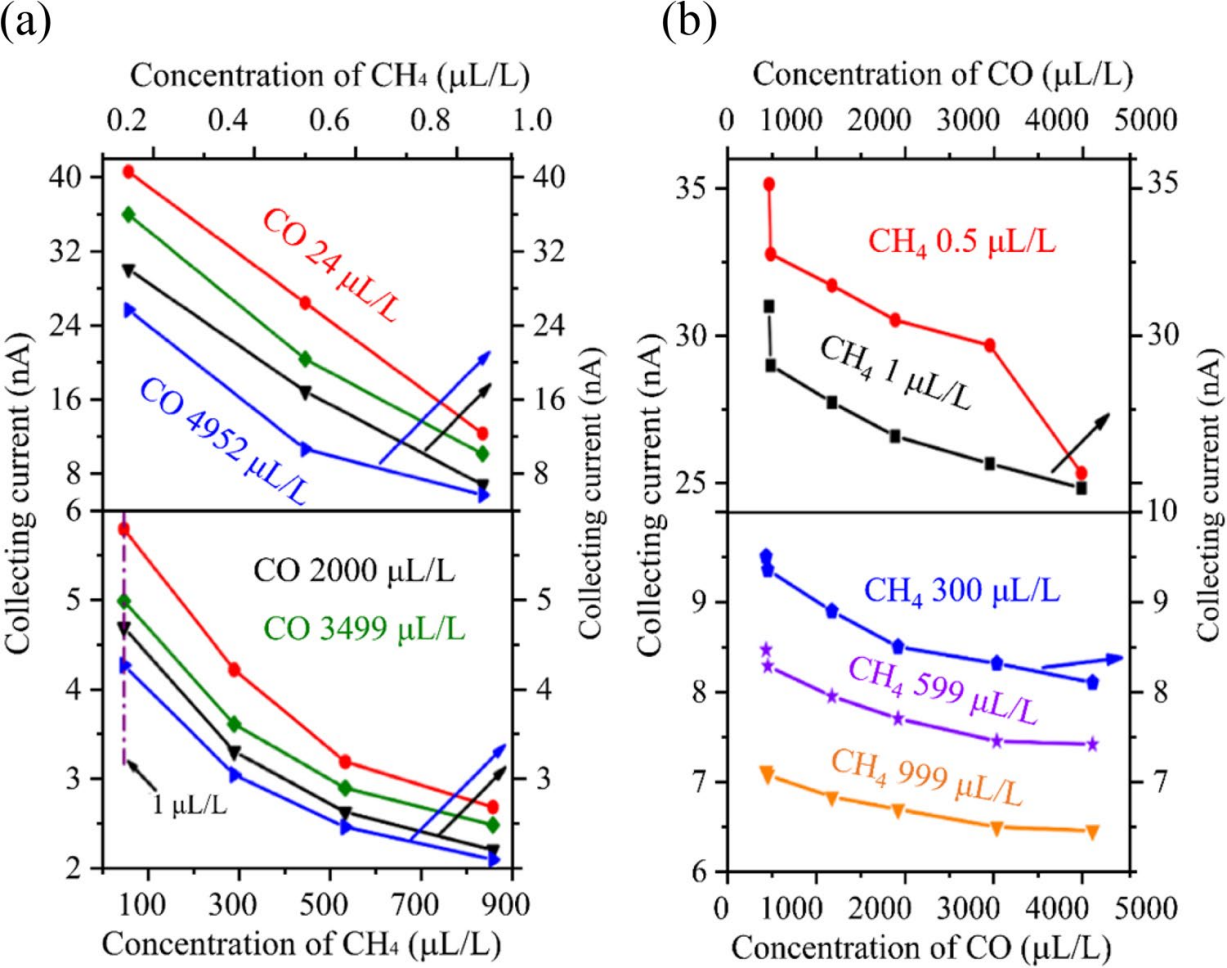
Detection of a CH_4_-CO mixture in N_2 _background using two different inter-electrode spacing sensors.** (a)** At 50°C, 120 V *U*_e_, and 1 V *U*_c_, the 100 μm sensor for the test of CH_4_ concentration in the rage of 0-1000 ppm and **(b)** 120 μm sensor for the detection of CO in the rage of 0-5000 μL/L.

Firstly, we fabricated two Φ = 1.2 mm sensors with *d* of 100 μm and 120 μm to detect CH_4_ (0-1000 ppm) and CO (0 - 5000 ppm) concentrations. At 50 °C, 120 V *U*_e_, and 1 V *U*_c_, the 100 μm sensor exhibited a maximum sensitivity of -378 nA/ppm at 0.5 ppm CH_4_, as shown in Fig. 8. The 120 μm sensor had a maximum sensitivity of -151.1 nA/ppm for 24 ppm CO but exhibited higher cross-sensitivity to CH_4_ (-7.09 × 10^-4^ ppm^-1^) compared to CO (-1.98 × 10^-4^ ppm^-1^), indicating a stronger CH_4_ effect on CO detection. We also tested H_2_-C_2_H_2_ gas mixtures using the same electrode spacing and compared these results with a Φ = 6 mm sensor array. The Φ = 1.2 mm sensor array demonstrated enhanced sensitivity (see Fig. S9), achieving − 345 nA/ppm at 5 ppm C_2_H_2_ and − 29 nA/ppm at 155 ppm H_2_, which was twice as high as the Φ = 6 mm sensors performance^11,14^.

**Figure 9:**
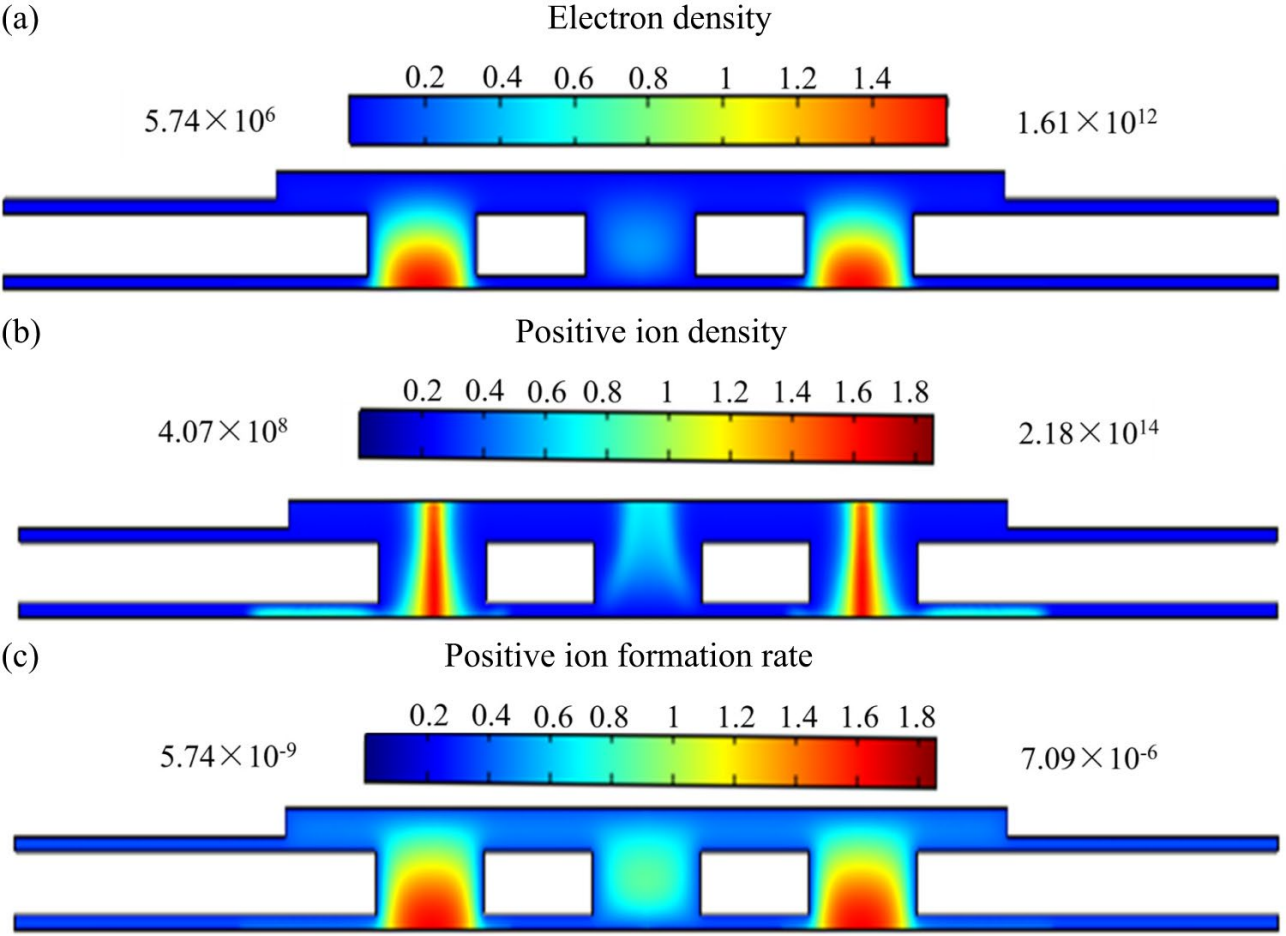
Gas discharge characteristics under RF excitation. **(a)** Electron density distribution, **(b)** electron density distribution and **(c)** positive ion formation rate of the sensor.

Secondly, we evaluated a three-sensor array (*d *= 75/100/120 µm) for detecting three-component mixtures using DC and RF power supplies. While DC detection mechanisms for CNTs cathode sensors were previously reported^11,14^, however, here our focus was on optimizing the performance of ionization sensor. Therefore, first we assessed the impact of RF and DC power supplies on the sensor’s discharge characteristics using a fluid model (see Fig. 2d). Simulation results as illustrated in Fig. 9, S10 and Table 4, we calculated the ionization characteristics such as the electron energy, *n*_e_, *n*_+_, *J*_c,_ and *J*_cathode_ distribution on the sensor’s cathode surface under DC and RF excitation modes. The results shows that under DC excitation, the positive ion density on the cathode surface steadily accumulates and eventually stabilizes. Under RF excitation, the ion density fluctuates with changes in the RF electric field’s direction and magnitude, with a gradual increase in peak density. This suggests that RF excitation induces positive ions and partially mitigates charge accumulation, although many ions oscillate between the electrodes due to the high frequency. Compared to DC excitation, RF excitation reduces cation density by about three orders of magnitude, minimizing ion bombardment and charge buildup on the cathode. This promotes discharge uniformity and stability, thereby improving the sensor’s hysteresis effects.

**Table 4:** Electrostatic field simulation results

Sensor structure	Supply	*n*_emax_ (1/m^3^)	*n*_+max_ (1/m^3^)	Electron Energy/eV	*j*_*c*_ (A/m^2^)	*j*_*cathode*_ (A/m^2^)
1.2 mm	RF	1.60 × 10^12^	2.18 × 10^14^	2.97	4.59 × 10^−7^	1.32 × 10^−7^
1.2 mm	DC	1.41 × 10^11^	8.57 × 10^13^	2.53	1.48 × 10^−7^	7.58 × 10^−8^

Then, we experimentally determined the impact of RF supply on discharge current on electrode separations in pure N_2_, with DC effects already reported previously^11,14^. Using a five-sensor array with electrode spacing from 60 μm to 120 μm, and maintaining conditions of 27 °C, 22.4% humidity, and even with wider spacing. Increasing RF power from 1 W to 6 W at 1.5 MHz resulted in a consistent rise in discharge current, from 51.21 nA at 1 W to 5123 nA at 6 W (Fig. S11). Due to system limitations, we chose 6 W power at 1.5 MHz for detecting trace SO_2_, NO, and O_2_.

**Figure 10:**
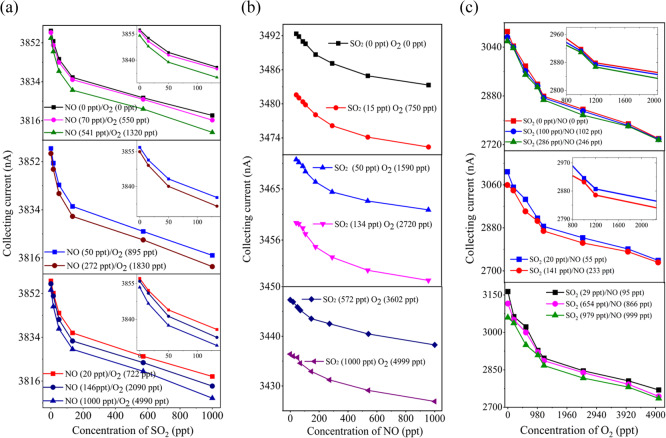
Simultaneous detection of trace concentration of a SO_2_/NO/O_2 _mixtures using radio frequency voltages (*U*_rf_) in N_2_. **(a)** At *U*_e_ 1.5 MHz 7 W, *U*_c_ 10 V, 27°C temperature, 22.4% RH, and 1 atm gas pressure, the collecting current of the 75 μm electrode spacing sensor decreases in single-value sensitivity as the SO_2_ concentration increases from 0-1000 ppt, **(b)** the collecting current of the 100 μm and **(c)** 120 μm electrode spacing sensors also decreases in a single-value sensitivity as the concentration of NO and O_2_, increases.

We fabricated a three-sensor array with 75 μm, 100 μm, and 120 μm electrode spacings for detecting SO_2_, NO, and O2. Using RF power at 1.5 MHz and 6 W for ppt-level detection (see Fig. S12) and DC voltage (*U*_c_ = 10 V, *U* _e_ = 120 V) for ppm-level detection (see Fig. S13). We found that the 75 μm sensor, tested at 27 °C, 22.4% humidity, and 1 atm, showed a consistent decrease in *I*_c_ with increasing SO_2_ concentration from 0 to 1000 ppt. It achieved a sensitivity of -445 pA/ppm at 2 ppt of SO2 (see Fig. 10a), surpassing its DC counterpart see Fig. S13C and 6 mm sensors^11,35^. This sensor also exhibited the highest cross-sensitivity of 2.65 × 10^-1^ nA/ppm^-1^, indicating significant interference from O_2_ and SO_2_ in NO detection (see Table 5). Similarly, the sensor with a *d* = 100 μm, *I*_c_ decreased consistently as the concentration increased from 1 ppt to 1000 ppt in Fig. 10b, it also demonstrated increased sensitivity of -605 pA/ppm to a concentration of 20 ppt of NO, surpassing the sensitivity of its DC power supply sensor (Fig. S13B) and the *Φ*= 6 mm sensor^11,35^. The sensor exhibited a strong influence of SO_2_ and O_2_ component with a cross-sensitivity coefficient of 2.23 × 10^−4^ nA/ppm^−1^ in NO detection, notably higher than that of the 75 μm sensor interference (Table 5). Moreover, the 120 μm sensor exhibited a -510 pA/ppm sensitivity to 10 ppt O_2_ (see Fig. 10c), outperforming its same sensor wit DC power supply sensitivity (Fig. S13A). It exhibited a consistent decrease in current with rising O_2_ concentrations from 1 ppt to 5000 ppt, though affected by SO_2_ and NO with a cross-coefficient of -2.42 × 10^-5^ nA/ppm^-1^. RF power significantly enhanced sensitivity and selectivity compared to DC as it evidenced in simulation results (Table 6). The 1.2 mm sensor was three orders of magnitude more sensitive than the 6 mm sensor^11–16,35^, demonstrating its effectiveness in detecting multiple gases at trace levels and supporting integrated sensor development.

**Table 5:** Maximum cross sensitivity of the sensors

S_CH4−CO_ _(max)_	S_CO−CH4_ _(max)_	S_H2−C2H2_ _(max)_	S_C2H2−H2_ _(max)_	S_SO2−NO−O2_ _(max)_	S_NO−SO2−O2_ _(max)_	S_O2−NO−SO2_ _(max)_
1.98 × 10^−2^	7.09 × 10^−3^	2.3 × 10^−4^	3.5 × 10^2^	-2.65 × 10^−1^	-2.23 × 10^−4^	-2.42 × 10^−5^

**Table 6:** Nanomaterial based three electrode sensor performance comparison matrix

Mechanism	Gas	Sensitivity S_*N*_ (ppm)	Response/recovery time (s)	Range (ppm)	Repeatability Error (%)	ref
Ionization	NO	-0.605	~ 7.12/4.6	0-1000 ppt	> 1	This study
Ionization	NO	12	< 40	0-250	> 2	^36^
Ionization	NO	0.53	90/900	0-5000	-	^37^
Resistive	NO	-	14/15	1-1000	-	^38^
Ionization	NO	-	8/7	0-1200	> 1.5	^12^
Ionization	SO_2_	-0.445	7/4	0-1000 ppt	3.1	This study
Absorption	SO_2_	> 0.98	10/30	0.5–15		^39^
Ionization	SO_2_	-	8/7	0-800	> 1.82	^16^
Resistive	SO_2_	0.018	97.12/93.91	3 ppm-500 ppb	~ 4	^40^
Ionization	SO_2_	2.3*10^−5^	140/130	0-100 ppm	-	^41^
Ionization	O_2_	-0.510	6.8/4.12	0-1000 ppt	2.3	This study
Resistive	O_2_	160	500/1000	0–40	-	^42^
Resistive	O_2_	5	37/40	5%	-	^43^
Ionization	O_2_	-0.03 pA/ppm	9/6	0-100000 ppm	-	^11^
Ionization	C_2_H_2_	-345	~ 7.3/4	0-2000	> 3	This study
Ionization	C_2_H_2_	6.81E-2	~ 100	0-1000	-	^43^
absorption	C_2_H_2_	1.06	-	20-100ppb	-	^44^
Ionization	C_2_H_2_	-124.74	9/6	0-198	2.6	^17^
Ionization	H_2_	-29.9	7.1/3.9	0-2800	> 2.4	This study
Ionization	H_2_	0.92	75	40-86ppb	-	^43^
Absorption	H_2_	0.35	15/72	20–300	-	^44^
Ionization	H_2_	-0.19	9/6	0-2005	4.9	^14^
Ionization	CH_4_	-151.1	7/4.1	1 ppb-25,000	~ 4	This study
Resistive	CH_4_	9.04	4/174	0-1000	-	^45^

### Technical assessment of the sensitivity, repeatability and response time

**Figure 11:**
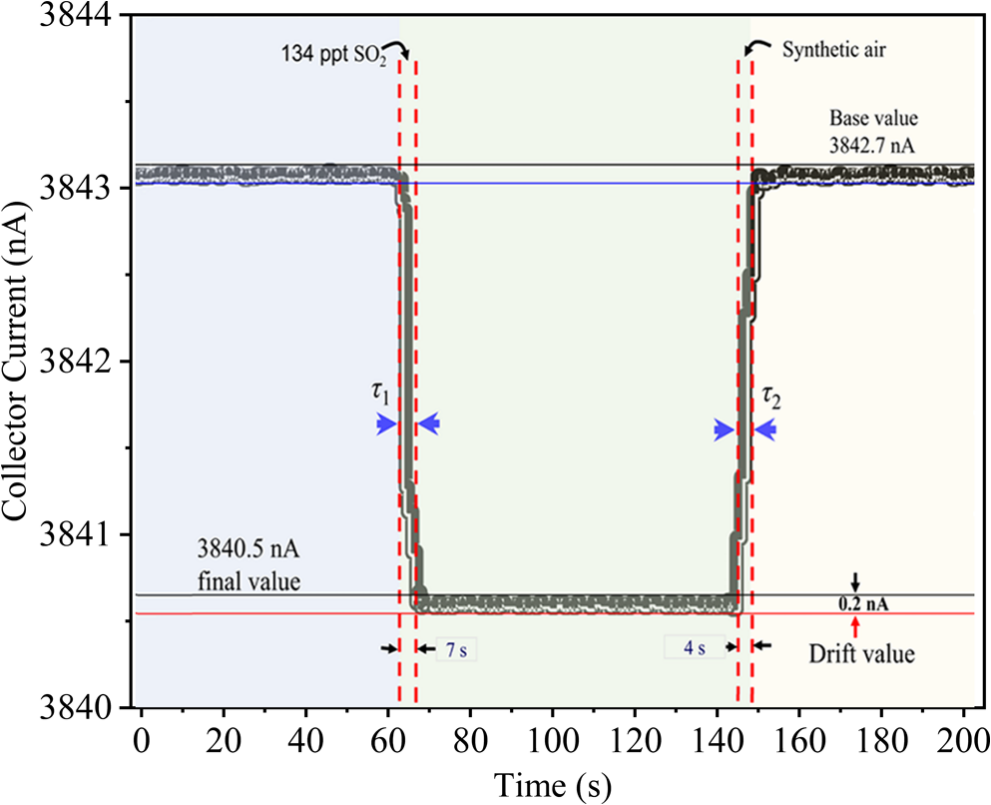
Response recovery time of the ionization based three-electrode novel gas sensor.

**Figure 12:**
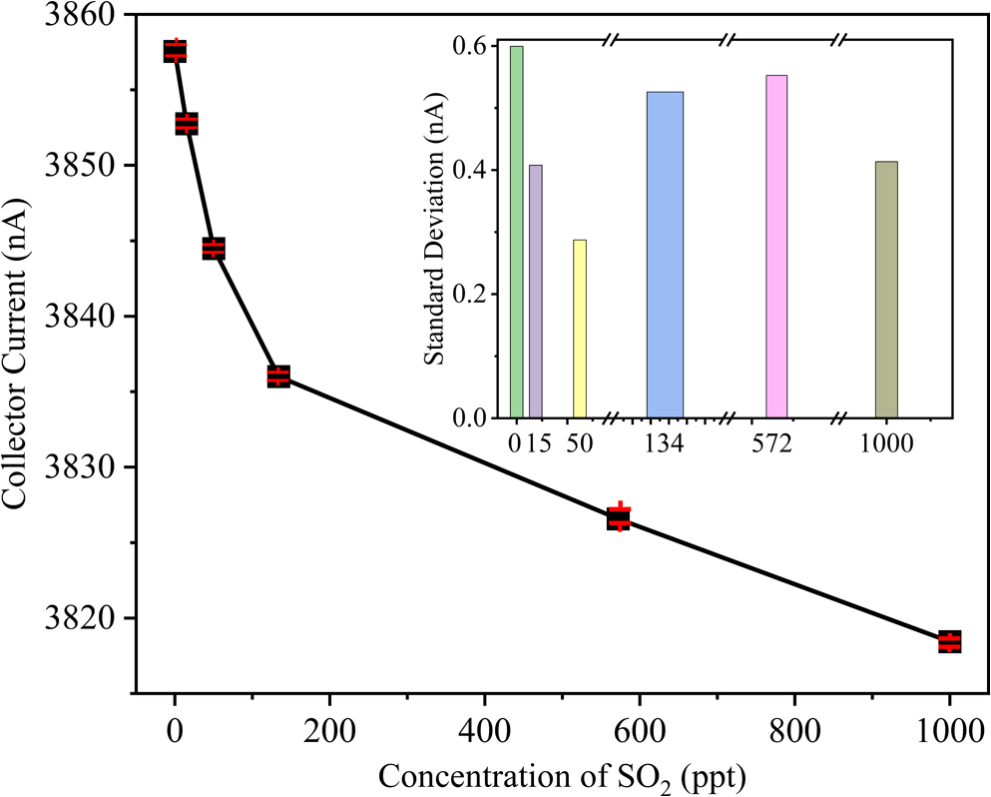
Collecting current vs measured curves of ten times repeated measurement of the 75 μm separation SO_2_ sensor.

The performance of the nanomaterial-based gas sensor is evaluated through the response/recovery time (see Fig. 11), sensitivity (see Fig. 10), linearity (see Fig. S14), and repeatability (see Fig. 12). Sensitivity *S* is calculated as *S*, *S* = Δ*I*_c_/Δ*ϕ*, for single gases, binary, and ternary mixtures. The three-electrode ionization sensor exhibits sensitivity that is approximately ∼ 2 – 3 orders of magnitude greater than other sensors (see Table 6).

Cross-sensitivity measures how gases affect each other’s readings. It quantifies the change in output per unit of interference, with *S*_i−j_ representing the impact of the *j*_th_ gas on *i*_th_ gas parameter, where *i*≠*j* and i, *j* ∈ {1,2,3} corresponding to SO_2_, O_2_, and NO.

13 $${S_{{\text{i-j}}}} = \frac{\Delta I_{\text{cmi}}}{I_{\text{cF.Si}} \times \Delta_{x\text{j}}}$$

Here, Δ*I*_cmSO2_ is the maximum deviation in the *I*_C_ of the *i*_th_, and *j*_th_ sensors, such as *ϕ*_SO2_ (e.g., *x*_1_) and Δ*x*_j_ is the change in the *j*_th_ parameter. *I*_cFSi_ denotes the maximum deviation between the highest and lowest *I*_C_ values of the *i*_th_ and *j*_th_ sensors due to the targeted parameter. For instance, when *i* and *j* refer to NO and O_2_ respectively, *I*_CFSO2_= *I*_CSO2_ − *I*_cNO_− *I*_cO2_. Smaller *S*_i−j_ values indicate stronger interference from the *j*_th_ parameter on the *i*_th_ sensor. For example, the maximum cross-sensitivity values are − 0.265 nA/ppm for SO_2_ with NO and O_2_, -2.23 × 10^−4^ for NO, and 2.42 × 10^−5^ for O_2_ (see Table 5).

We performed repeatability experiments 10 times (see Fig. 12) and assessed the relative error (*r*), standard deviation (σ_j_), and repeatability error (*δ*_R_) for the novel sensor (see Table 6) according to the relevant standards^14^. The results demonstrate that all three sensors exhibit impressively low relative and repeatability errors, both below 3.1% (refer to Table 6), indicating excellent repeatability. In Fig. S14, the sensitivity curves were fitted using various algorithms, including logarithmic, cubic, and quadratic functions. The logarithmic fitting model yielded the lowest mean squared error (MSE) compared to the others.

In gas detection, timing metrics are crucial. This study measured the response time (τ_1_) and recovery time (τ_2_) of a new triple-electrode ionization sensor. The sensor responded in 7 s and recovered in 4 s when switching from synthetic air to an SO_2_ mixture, with these times representing *t*_90_ and *t*_10_ (see Fig. 11). This quick response underscores the sensor’s superior adaptability and performance compared to our previous and other sensors (see Table 6).

As no other research closely matches our work on sensor arrays, we compared the performance of our proposed sensors with existing single-gas and mixture detectors (see Table 6). Our sensor’s sensitivity is 2 – 3 orders of magnitude greater than that of others, and it exhibited the smallest linearity error, relative error and repeatability errors no more than 4.1%, 2.9% and 3.1% respectively. The triple-electrode ionization sensor array offers a low detection limit for multiple gases, high sensitivity, and improved integration.

## Conclusion

The present research addressed the issue of positive ion bombardment in ionization gas sensors, which adversely affects their lifespan, current collection, and detection accuracy. We developed a detailed electric field distribution and discharge simulation model to investigate how electrode structure, cathode material, DC and RF voltage, and temperature influence sensor performance. The simulation findings were validated through experimental testing.

Our results revealed that reducing the diameter of the diffusion aperture increased the reverse electric field (*E*_1_) near the cathode nanotips. In contrast to previous sensor, this enhancement accelerates approximately two-thirds of positive ions in a 2 × 6 mm sensor structure, leading to non-self-sustaining discharges. Consequently, this modification not only mitigates positive ion bombardment but also improves the sensor’s current collection. Simulation analysis of eight different structures indicated that the Φ = 1.2 × 9 mm sensor best retains *E*_1_ around the nanotips, thereby improving current density compared to other structures.

We also evaluated CNTs, graphene, and Au as cathode materials, finding that Au-cathode sensor outperformed the others. Gold cathodes exhibited approximately twice the maximum electron emission rate (*n*_emax_), positive ion density (*n*_+_), collecting current, and cathode current density compared to the other materials. Additionally, gold sensors demonstrated higher sensitivity of -9.6 pA/ppm to 13 ppm of H_2_ gas and superior oxidation resistance, maintaining stable performance even in 100% O_2_ environments, unlike graphene and CNTs. We also investigated how nanotip height and spacing affect electric field intensity. Our findings indicate that the enhancement factor increases with nanotip height but plateaus when the spacing exceeds twice the height. Experimental results showed that sensor performance varies with temperature and electric field, following the field-assisted thermal emission law. RF excitation significantly improved sensor performance compared to DC excitation by reducing cation density and minimizing ion bombardment.

The sensor array exhibited a rapid response time of 7 s and a recovery time of 4 s. It demonstrated exceptional sensitivity, 2–3 orders of magnitude greater than other sensors with highest range of ppt level, and the smallest linearity error, with relative and repeatability errors not exceeding 4.1%, 2.9%, and 3.1%, respectively.

## Supplementary Information

Supplementary Material 1

## Data Availability

The authors confirm that the data supporting the findings of this study are available within the article. The data that support the findings of this study are available on request from the corresponding authors, [Muhammad Waqas] [Yong. Zhang], upon reasonable request.
