# Supplementary material for: Structural design and temperature control enabling high sensitivity nanomaterial-based three-electrode gas sensors
Source: Sci Rep. 2025 Jul 1;15:20487. doi: 10.1038/s41598-024-76678-2 (PMC12215397; doi:10.1038/s41598-024-76678-2)
Supplement: Supplementary file 1 — Supplementary Material 1 [file 41598_2024_76678_MOESM1_ESM.docx]

**Supplementary information**

**Structural design and temperature control enabling high sensitivity of nanomaterials based three electrodes gas sensor**

Muhammad, Waqas^1,2, *^, Yong. Zhang^1,3, *^, Saif Aldeen Saad Obayes. Alkadhim^1,2^, Xiaoyu. Li^1,2^, Liang. Xie^1,2^

^1^State key Laboratory of Electrical Insulation and Power Equipment Xi’an Jiaotong University, 28 Xianning West Road, Xi’an, 710049, P. R. China.

^2^School of Electrical Engineering, Xi’an Jiaotong University, 28 Xianning West Road, Xi’an, 710049, P. R. China.

^3^School of Instrument Science and Technology, Xi’an Jiaotong University, 28 Xianning West Road, Xi’an, 710049, P. R. China.

*Correspondence and requests for materials to be addressed to

Y.Z. and M.W ([zhyong@mail.xjtu.edu.cn](mailto:zhyong@mail.xjtu.edu.cn), vik_hashmi89@stu.xjtu.edu.cn)

1. Senor MEMS process and fabrication

(A) (B)


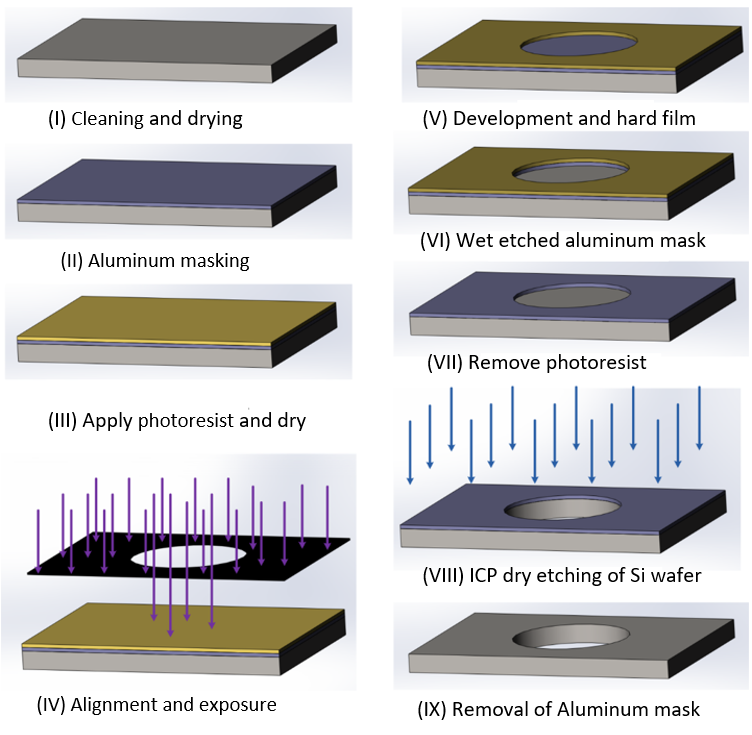

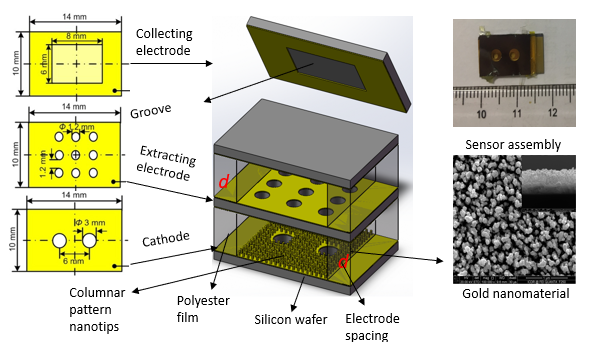


**Fig. s1. Fabrication process of three electrode ionization sensor**. (A) MEMS process (B) Schematic of a 1.2 × 9 mm sensor. As depicted in the figure, the sensor's electrodes are fabricated using MEMS technology, which involves sputtering, coating, and rapid annealing on a single-crystal silicon wafer. The silicon microstructures on the cathode are created through deep silicon etching. Au/Ni/Ti is used for the insulating strips, which determine the electrode spacing (*d*) of the sensor. The electrodes are then assembled and connected using insulating glue and strips to form the complete sensor.

**Table. s1.** Chemical reactions

| No | Reaction | Reaction rate coefficient  *k*_r_/m^3^ s^-1^ | Supplementary  ref |
| --- | --- | --- | --- |
| R1 | $\text{e+N}_{2}\to\text{N}_{2}\text{+e}$ | *f* (*ε*) | [1] |
| R2 | $\text{e+N}_{2}\to\text{N}_{2}\text{(}A{}^{3}\Sigma_{\text{u}}^{+}\text{)+e}$ | *f* (*ε*) | [1] |
| R3 | $\text{e+N}_{2}\to\text{N}_{2}\text{(}\text{a}'^{1}\Sigma_{\text{u}}^{-}\text{)+e}$ | *f* (*ε*) | [1] |
| R4 | $\text{e+N}_{2}\to\text{N}_{2}^{+}\text{+e+e}$ | *f* (*ε*) | [1] |
| R5 | $\text{e+N}_{4}^{+}\to\text{N}_{2}\text{+N}_{2}$ | *f* (*ε*) | [1] |
| R6 | $\text{e+e+N}_{2}^{+}\to\text{N}_{2}\text{+e}$ | *f* (*ε*) | [1] |
| R7 | $\text{e+N}_{2}^{+}\text{+N}_{2}\to\text{N}_{2}\text{+N}_{2}$ | 3.12×10^-23^*T*_e_^-1.5^ | [2] |
| R8 | $\text{N}_{2}\text{(}A{}^{3}\Sigma_{\text{u}}^{+}\text{)+N}_{2}\text{(}\text{a}'{}^{1}\Sigma_{\text{u}}^{-}\text{)}\to\text{e}+\text{N}_{2}\text{+N}_{2}^{+}$ | 5×10^-11^ | [2] |
| R9 | $\text{N}_{2}\text{(}\text{a}'{}^{1}\Sigma_{\text{u}}^{-}\text{)+N}_{2}\text{(}\text{a}'{}^{1}\Sigma_{\text{u}}^{-}\text{)}\to\text{e}+\text{N}_{2}\text{+N}_{2}^{+}$ | 2×10^-10^ | [2] |
| R10 | $\text{N}_{2}\text{(}A{}^{3}\Sigma_{\text{u}}^{+}\text{)+N}_{2}\text{(}\text{a}'{}^{1}\Sigma_{\text{u}}^{-}\text{)}\to\text{e+N}_{4}^{+}$ | 5×10^-11^ | [3] |
| R11 | $\text{N}_{2}\text{(}\text{a}'{}^{1}\Sigma_{\text{u}}^{-}\text{)+N}_{2}\text{(}\text{a}'{}^{1}\Sigma_{\text{u}}^{-}\text{)}\to\text{e+N}_{4}^{+}$ | 2×10^-10^ | [3] |
| R12 | $\text{N}_{2}\text{(}A{}^{3}\Sigma_{\text{u}}^{+}\text{)+N}_{2}\to\text{N}_{2}\text{+N}_{2}$ | 3×10^-18^ | [4] |
| R13 | $\text{N}_{2}\text{(}\text{a}'{}^{1}\Sigma_{\text{u}}^{-}\text{)+N}_{2}\to\text{N}_{2}\text{+N}_{2}$ | 1.9×10^-13^ | [4] |
| R14 | $\text{N}_{2}^{+}\text{+N}_{2}+\text{N}_{2}\to\text{N}_{4}^{+}\text{+N}_{2}$ | 1×10^-29^(300/*T*_g_) | [4] |
| R15 | $\text{N}_{4}^{+}\text{+N}_{2}\to\text{N}_{2}^{+}\text{+N}_{2}\text{+N}_{2}$ | 2.1×10^-10^exp (*T*_g_/121) | [4] |
| R16 | $\text{N}_{2}\text{(}A{}^{3}\Sigma_{\text{u}}^{+}\text{)+NO}\to\text{N}_{2}\text{+NO}$ | 6.4×10^-11^ | [5] |
| R17 | $\text{N}_{2}\text{(}\text{a}'{}^{1}\Sigma_{\text{u}}^{-}\text{)+NO}\to\text{N}_{2}\text{+N+O}$ | 4×10^-10^ | [6] |
| R18 | $\text{N}_{2}\text{(}A{}^{3}\Sigma_{\text{u}}^{+}\text{)+SO}_{2}\to\text{N}_{2}\text{+SO+O}$ | 6.2×10^-10^ | [6] |
| R19 | $\text{N}_{2}\text{(}A{}^{3}\Sigma_{\text{u}}^{+}\text{)+O}_{2}\to\text{N}_{2}\text{+O}_{2}$ | 1.3×10^-12^ | [6] |
| R20 | $\text{N}_{2}\text{(}A{}^{3}\Sigma_{\text{u}}^{+}\text{)+O}_{2}\to\text{N}_{2}\text{+O}_{2}$ | 1.3×10^-12^ | [2] |
| R21 | $\text{N}_{2}\text{(}A{}^{3}\Sigma_{\text{u}}^{+}\text{)+O}_{2}\to\text{N}_{2}\text{+O+O}$ | 5×10^-18^exp(-210/*T*_g_) | [1] |
| R22 | $\text{N}_{2}\text{(}A{}^{3}\Sigma_{\text{u}}^{+}\text{)+CO}_{2}\to\text{N}_{2}\text{+CO+O}$ | 2.0×10^-14^ | [1] |
| R23 | $\text{N}_{2}\text{(}A{}^{3}\Sigma_{\text{u}}^{+}\text{)+CO}\to\text{N}_{2}\text{+CO}$ | 1.6×10^-12^ | [1] |
| R24 | $\text{N}_{2}^{\text{+}}\text{+NO}\to\text{N}_{2}\text{+NO}^{\text{+}}$ | 3.9×10^-16^ | [1] |
| R25 | $\text{N}_{4}^{\text{+}}\text{+NO}\to\text{N}_{2}\text{+N}_{2}\text{+NO}^{\text{+}}$ | 3.9×10^-16^ | [5] |
| R26 | $\text{N}_{2}^{+}\text{+SO}_{2}\to\text{SO}_{2}^{+}\text{+N}_{2}$ | 6.2×10^-10^ | [6] |
| R27 | $\text{N}_{2}^{+}\text{+CO}\to\text{CO}^{+}\text{+N}_{2}$ | 7×10-10 | [6] |
| R28 | $\text{N}_{2}^{+}\text{+CO}_{2}\to\text{CO}_{2}^{+}\text{+N}_{2}$ | 9×10-10 | [1] |
| R29 | $\text{N}_{2}^{+}\text{+O}_{2}\to\text{O}_{2}^{+}\text{+N}_{2}$ | 1.04×10^-9^Tg^-0.5^ | [4] |
| R30 | $\text{N}_{4}^{+}\text{+O}_{2}\to\text{O}_{2}^{+}\text{+2N}_{2}$ | 2.4×10^-10^ | [4] |
| R31 | H_2_ + H_2_^+^→ H + H_3_^+^ | 2×10^−15^ | [1] |
| R32 | H_2_ + N_2_ + H^+^ → N_2_ + H_3_^+^ | 3.1×10^−41^ | [1] |
| R33 | H_2_ + N_4_^+^ → N_2_ + N_2_ +H_2_^+^ | 3×10^−16^exp(−1800/*T*_g_) | [1] |
| R34 | N_2_ (A^3^∑_u_^+^) + H_2_ → H + H + N_2_ | 4.4×10^−16^exp(−3500/*T*_g_) | [7] |
| R35 | N_2_ (a′^1^∑_u_^−^) + H_2_ → H + H + N_2_ | 2.6×10^−17^ | [7] |
| R36 | H + H + N_2_ → H_2_ + N_2_ | 1.8×10^−42^/*T*_g_ | [8] |
| R37 | N_2_ (A^3^∑_u_^+^) + C_2_H_2_ → C_2_H + H + N_2_ | 2×10^−16^ | [7] |
| R38 | N_2_(a′^1^∑_u_^−^)+C_2_H_2_→C_2_H+H+N_2_ | 3×10^−16^ | [7] |
| R39 | C_2_H_2_ + H → C_2_H + H_2_ | 1×10^−16^exp(−14000/*T*_g_) | [9] |
| R40 | C_2_H_2_ + e → C_2_H + H + e | 1.0×10^−16^ | [9] |


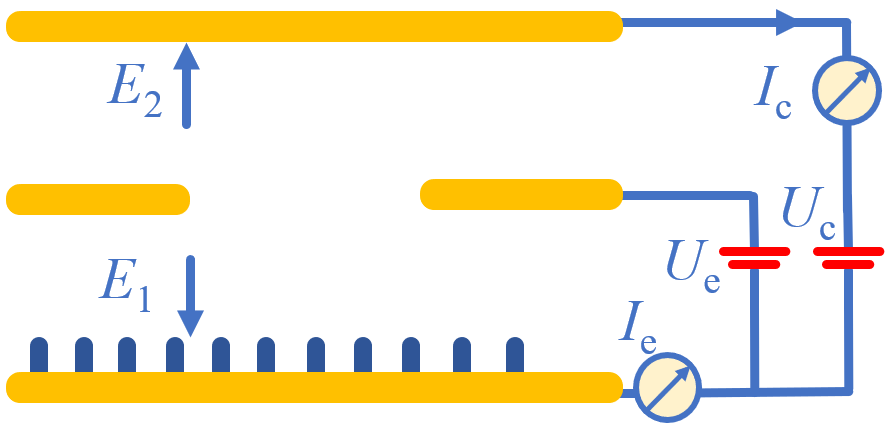


**Fig. s2.** Schematic diagram of the supply system to the sensor electrode, where *U*_e_ > *U*_c_ than, which offers two electric fields *E*_1_ and *E*_2_ in reversed field direction.

1. Simulation results on effects of different electrodes structure on sensor performance

*
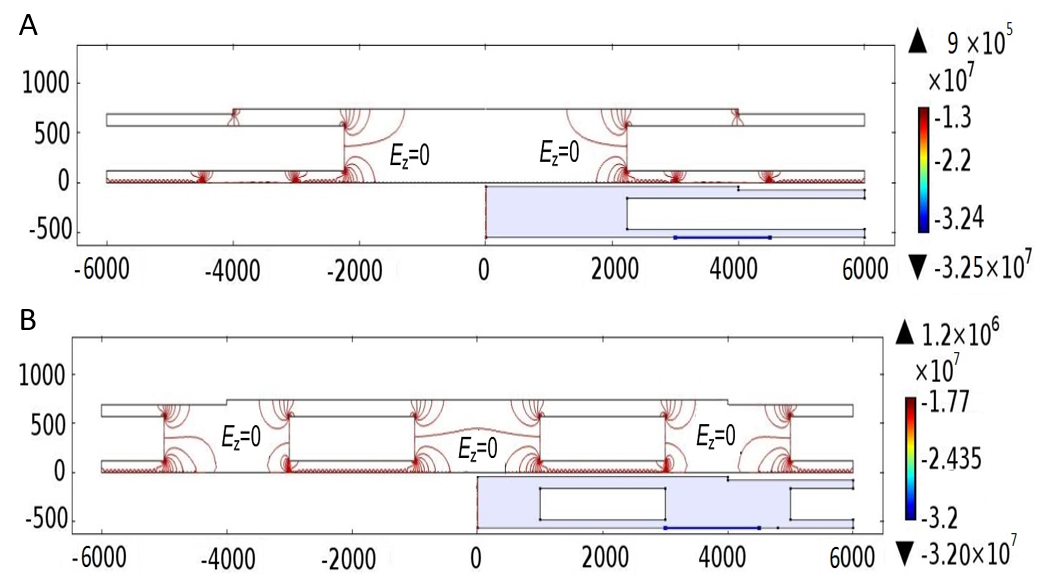
*

**Fig. s3.** Electric field distribution in the 6 mm and 2 mm sensor in length direction. To differentiate the difference of fields distribution, we draw a line (*E*_z_) from the center of the sensors. The *E*_z_ on each line is equal, and the line with *E*_z_=0 has been plotted in the (A) and (B) for 6 mm and 2 mm sensors respectively. We found that for the 6 mm configuration sensor, there was no reverse electric field in the center, however, only the edges of the extracting diffusion aperture have reverse electric. While for a 2 mm diameter sensor, a reverse electric field is present throughout the collecting electrode.


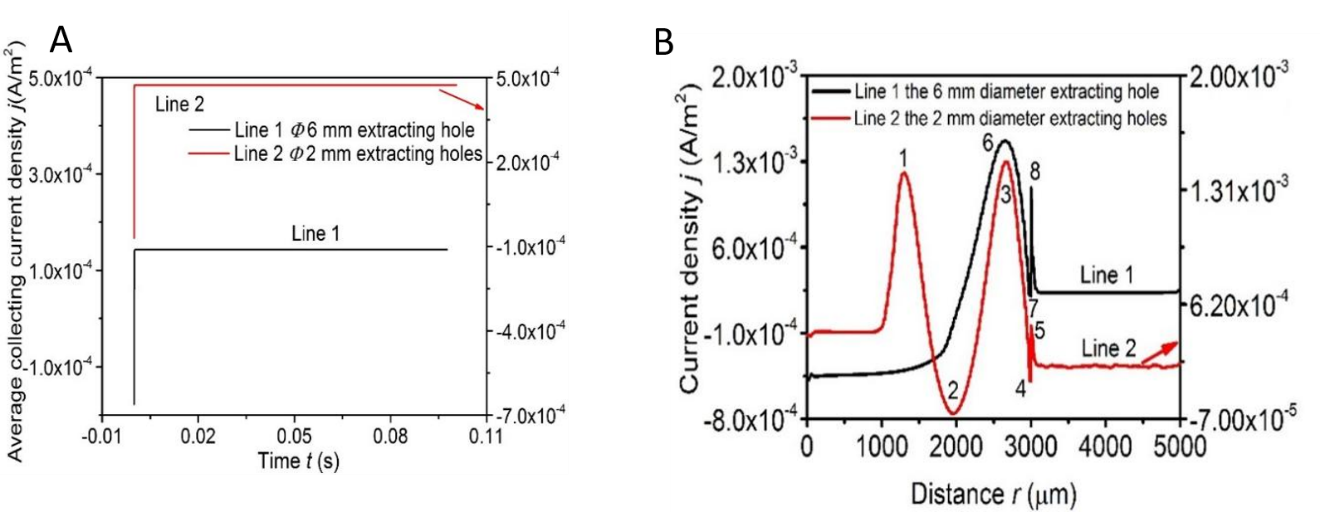


**Fig. s4.** The difference of distribute current density in the 6 mm and 2 mm sensors from the center of the electrodes to the edges. **(A)** The lines 1 and 2 were plotted corresponding to Fig. 3a and 3b, respectively. Where collecting current density of 2mm sensor is higher than 6 mm sensor. **(B)**  The Peaks 1, 2, 3, 4 and 5 are the pulses of the current density of 2mm sensor (line 2) throughout the sensor structure, which are at 1.41×10^-3^ A/m^2^, 1.48×10^-3^ A/m^2^, 4.92×10^-4^ A/m^2^, -3.74×10^-5^ A/m^2^, 1.60×10^-4^ A/m^2^, and 6, 8, and 7 are the pulses of current density of 2mm sensor (line 2), which are 1.47×10^-3^ A/m^2^, 1.09×10^-3^ A/m^2^, and 2.11×10^-4^ A/m^2^. Due to the collection of positive ions at collecting groove, we also found some pulses 4, 5 and 7, 8 at 3000 μm. Since there is no reverse electric field in the middle of the extracting aperture of line 1, there is a peak value 6 at the edge of the extracting aperture. Due to the small area without reverse electric field in the middle of the extracting aperture of line 2, there are peaks 1 and 3 at the edge of the extracting hole, and a lower peak at 2 in the middle. Overall, the current density of 2mm model remained two times higher than 6 mm model.


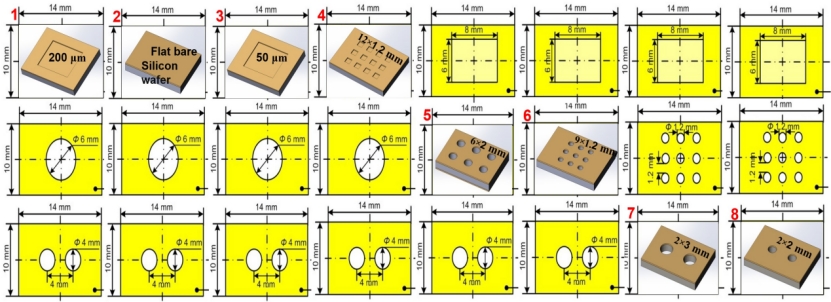


**Fig. s5.** The morphology of eight sensor structure. The sensors electrodes are same in the size (10×14 mm) however different in collecting, extracting and cathode electrodes diffusion holes and groves.

1. Simulation results on effects of different nano-material on sensor performance

**Table. S2.** Ionization characteristics of CNTs, graphene and gold nanomaterial cathode sensor

| Ionization  characteristics | Au-cathode | Graphene cathode | CNTs cathode |
| --- | --- | --- | --- |
| *n*_emax_ (1/m^3^) | 1.86×10^12^ | 1.73×10^11^ | 3.21×10^11^ |
| *n*_+max_ (1/m^3^) | 2.54×10^6^ | 1.13×10^5^ | 1.12×10^5^ |
| *j*_c_ (A/m^2^) | 3.47×10^-3^ | 1.16×10^-4^ | 4.98×10^-4^ |
| *j*_cathode_ (A/m^2^) | 3.81×10^15^ | 3.05×10^15^ | 3.24×10^15^ |


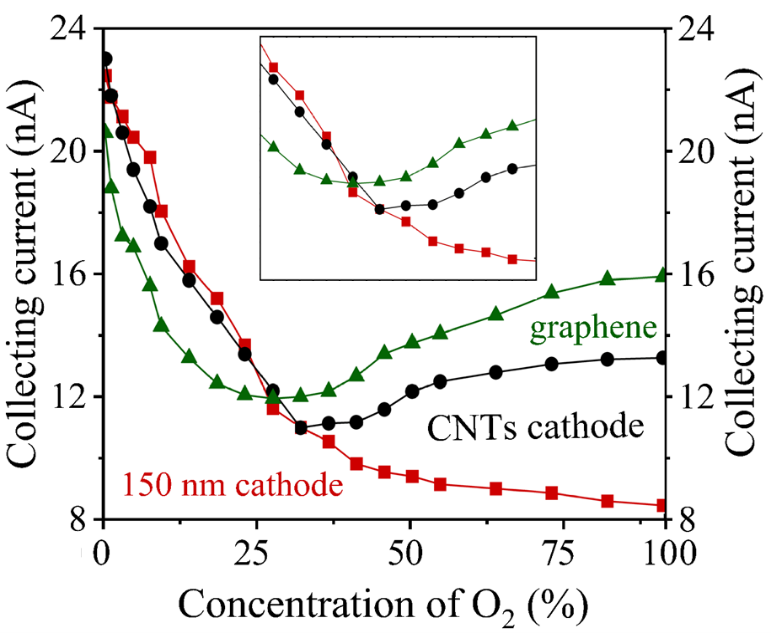


**Fig. s6.** The performance comparison of Ag-CNTs, pure CNTs and Au nanomaterial cathode sensors in protecting against corrosion damage using a *Φ* = 1.2 mm sensor with 100% of O_2_ concentration. At 75°C, 250 V *U*_e_, 10 V *U*_c_, and 120 μm electrode spacing, under continuous charged measurement with 100% O_2_ concentration, the 100 nm Ag-CNTs cathode sensor was destroyed at 30% oxygen concentration, and the bare carbon tube sensor was damaged at 33% concentration, demonstrating multi-value sensitivity, however the 150 nm Au nanomaterial cathode sensor showed no damage after exposure to 100% O_2_.

1. Simulation results on emission current of sensor in varying temperature, voltages and nanotips morphology

The three-electrode sensor follows the field-assisted thermal emission law; temperature and electric field affects the emission current of the sensor. We found collecting current I_c_ is exponentially dependent on gas temperature T and applied voltages in pure (see Fig. 7a and 7b main text). Hence, the relationship between the logarithm lnje of current density je and the -1000/T of temperature T and average electric field is obtained in supplementary Table. S3. Where je = I_e_/S = ~2I_c_/S. S is the total cross-sectional area of the Au-thin film. It can be seen from the Fig. 7a and 7b in the main text that the emission characteristics of gold nanostructured three-electrode sensors at different induced electrode voltages and temperatures are different.

The relationship between the cathode current density j_e_ and the extracted electrode voltage and temperature of the gold nanostructured gas sensor can be calculated. The cathode of the experimental gold nanostructure sensor is 14 ×10 mm (Fig. S1B main text), with the cathode aperture of 2× Φ3 mm, and the width of the two insulating strips is about 4 mm, then the effective area of the gold film cathode was derived from the equation S1, as shown in Fig. S7A. Assume that the array of gold nanotips is evenly arranged with a radius r of 25 nm and an average spacing of 300 nm, the S_grid_ is a region occupied by a single gold nanotip with a S_grid_ = 300 × 300 nm. The effective area of cathode gold thin films S_AuF_ was calculated from equations S3.


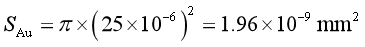
 (S1)


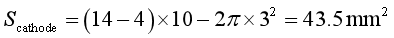
 (S2)


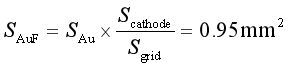
 (S3)

Where *S*_Au_ is the effective area of a single nanotip. The cathode current density *j*_e_ is equal to the induced current *I*_e_ divided by the area of the gold nanostructured film *S*_AuF_, which was obtained by the formula S4.


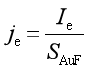
 (S4)

The results of *E*_1/2_ and *lnj_e_* at different induced electrode voltages are shown in Table. S3, and the results *of −1/T* and *lnj*_e_ at different temperatures are shown in Table S4, indicating that the *lnje* of the sensor is linearly correlated with *E*_1/2_ and *−1/T*, respectively, which is in line with the law of field-assisted thermal emission ^11 (main text)^.


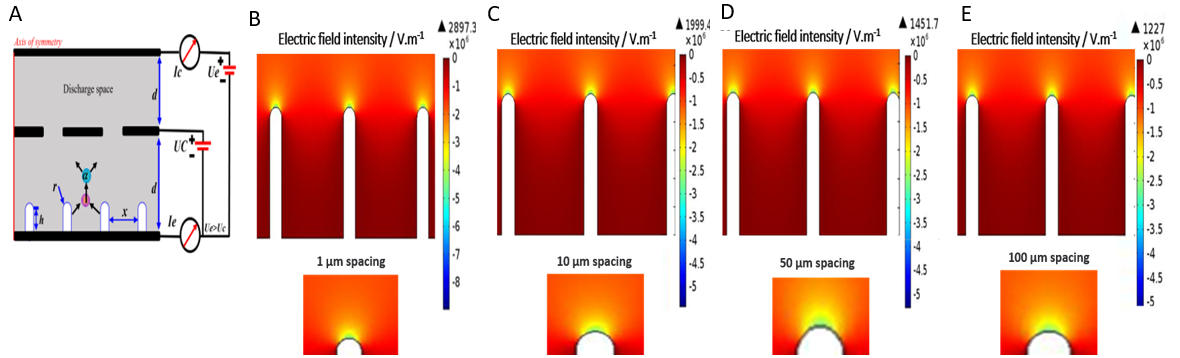


**Fig s7.** The effects of spacing (*x*) between cathode nanotips on the field enhancement factor (γ). **(A)** At fixed *r* = 25 nm and *h* = 300 nm, we set the *U*_cathode_ boundary electric potential at 0 V and when the electrode spacing is 1 μm, 10 μm, 50 μm, and 100 μm, the extracting electrode boundary voltages *U*_e_ are at 1 V, 10 V, 50 V, and 100 V, respectively, and *U*_c_ at 1 V, we calculated the electric field intensity (*E*_av_) and the (γ) field enhancement factor in discharge space using the finite element method **(B)** At *x*= 1 μm, the electrostatic field intensity of the gold nanostructure array model is about 8.96 V/μm and the field enhancement factor is γ=8.96. **(C)** At *x*= 10 μm, the electric field intensity decreased to *E*_av_ = 5.44 V/μm and so to γ=5.44. **(D)** At *x*= 50 μm, *E*_av_ = 5.28 V/μm and the γ=5.28. **(E)** At *x*= 100 μm, *U*_e_ = 100 V, the *E*_av_ = 5.25 V/μm and the γ=5.25.


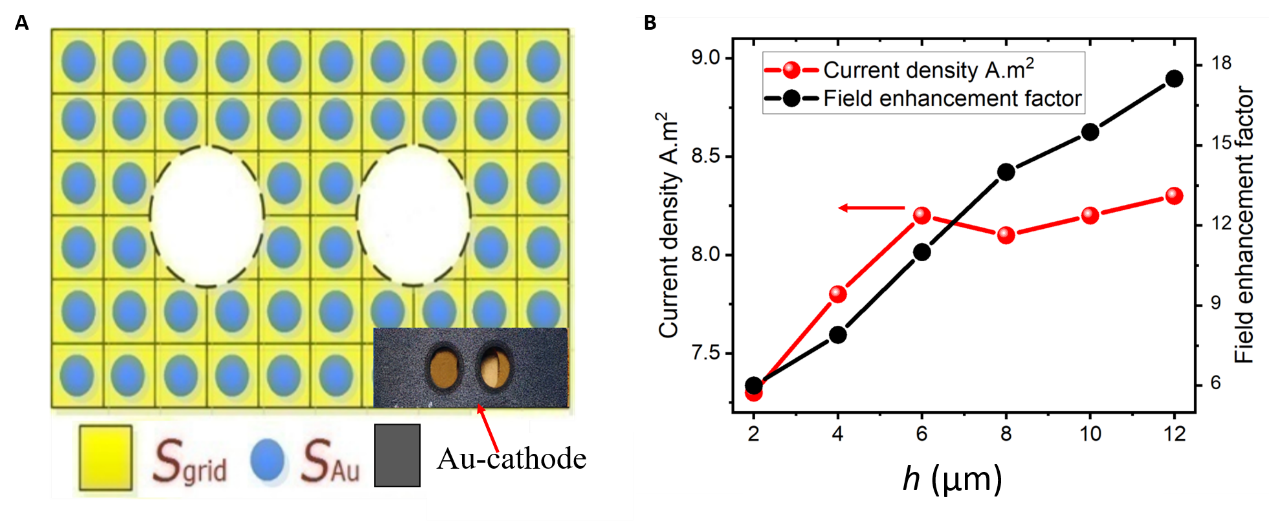


**Figure s8.** The impact of emission current on a 150 nm gold nanostructure cathode sensor. **(A)** Illustrates the actual area of the 150 nm Au-cathode. **(B)** The relationship between height (*h*) and the field enhancement factor of the gold nanomaterial is explored. It is observed that as the height of the Au-nanostructured material increases from 2 μm to 12 μm, both the cathode current density and field enhancement factor rise linearly. However, at a height of 6 μm, the cathode current density begins to decline, indicating that beyond 600 nm of height, further increases have no significant effect on cathode current density.

**Table s3.** Calculated results of E_1/2_ and lnj_e_ at different temperature.

| T/℃ | *I*_c_/nA | -1/T (K^-1^) | *l*nj_e_/A∙m^-2^ |
| --- | --- | --- | --- |
| 30 | 4.992 | -3.30E-03 | -7.47 |
| 35 | 6.617 | -3.25E-03 | -7.19 |
| 40 | 10.204 | -3.19E-03 | -6.76 |
| 45 | 17.327 | -3.14E-03 | -6.23 |
| 50 | 30.348 | -3.09E-03 | -5.67 |
| 55 | 53.698 | -3.05E-03 | -5.09 |
| 60 | 95.858 | -3.00E-03 | -4.52 |
| 65 | 157.592 | -2.96E-03 | -4.02 |
| 70 | 241.009 | -2.91E-03 | -3.59 |

**Table s4.** Calculated results of E_1/2_ and lnj_e_ at different voltages.

| *U*_e_/V | *I*_c_/nA | *E*_1/2_/V∙m^-1^ | *l*nj_e_/A∙m^-2^ |
| --- | --- | --- | --- |
| 50 | 9.723 | 1.96E+03 | -6.80 |
| 80 | 15.904 | 2.48E+03 | -6.31 |
| 100 | 20.524 | 2.77E+03 | -6.06 |
| 130 | 27.271 | 3.16E+03 | -5.77 |
| 150 | 30.629 | 3.39E+03 | -5.66 |
| 180 | 36.536 | 3.72E+03 | -5.48 |
| 200 | 39.686 | 3.92E+03 | -5.40 |
| 230 | 42.671 | 4.20E+03 | -5.32 |
| 250 | 47.979 | 4.38E+03 | -5.21 |

1. **Experimental results on two gases mixtures detection**


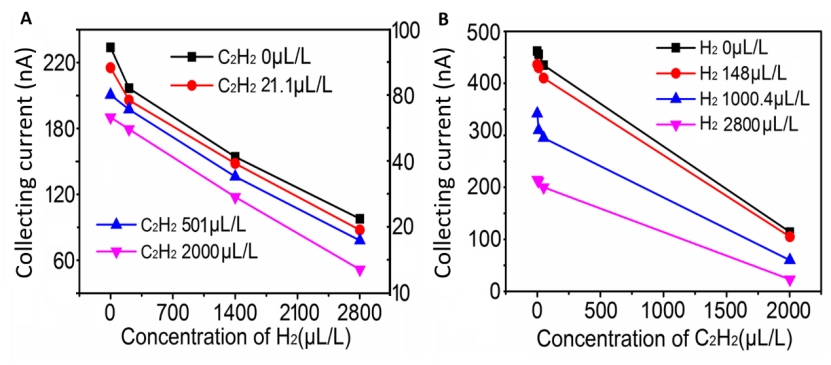


**Fig. s9.** Sensitivity characteristics of the H_2_-C_2_H_2_ mixtures in N_2_ background. **(A)** The H_2_ sensor with 100 μm electrode spacing shows a single value decrease collecting current as the concentration of H_2_ sensor increased from 0-2800 ppm. **(B)** The 120 μm sensor also shows the decrease collecting current as the concentration of C_2_H_2_ gas increase from 0 to 2000 ppm. The sensitivity was measured at different points when the mixture gas being constant and measured gas concentration increase, the sensors show higher sensitivity then other sensors. At *U*_e_ of 120 V and *U*_c_ of 5 V, we recorded maximum sensitivity of 120 μm sensor at -345 nA/ppm to a 5 ppm concentration of C_2_H_2_ gas. However, the maximum sensitivity of the 100 μm sensor remained at 29.9 nA/ppm for a 155 ppm concentration of H_2_ gas, which is lower than the sensitivity of the 120 μm sensor. Overall, the sensitivity of the C_2_H_2_ and H_2_ sensors is higher than that of the H_2_ and C_2_H_2_ sensors with a 6 mm structure in ref ^11,16 (main text)^.

1. Simulation results gas discharge using DC power supply


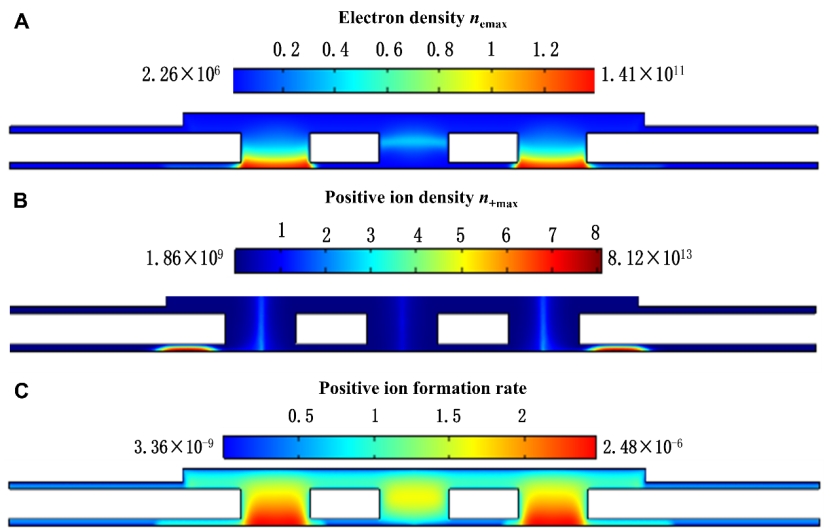


**Fig. s10**. Gas discharge characteristics under DC excitation. **(A)** electron density distribution (*n*_e_), **(B)** electron density distribution (*n*_e_) and **(C)** electron density distribution (*n*_e_) of the sensor.

1. Effects of power and frequency on electrode spacing


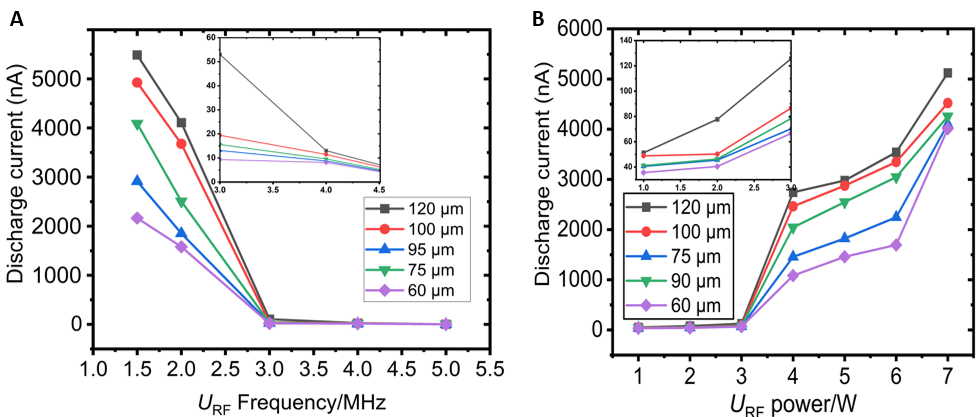


**Fig. s11.** Effects of RF power and frequency on electrode spacing of the sensor. **(A)** The response of sensor’s array to varying power frequency. As the power frequency increases from 1.5 MHz to 5.0 MHz, exhibiting a decrease in discharge current. **(B)** The response of sensor’s array to varying power at a fixed frequency of 1.5 MHz, an increase in RF power from 1 W to 7 W results in a continuous increase in discharge current.

1. **Experimental results on three gases mixtures detection**


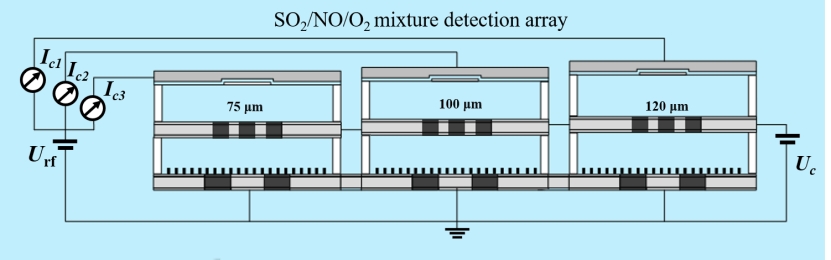


**Fig. s12.** The array of three sensors with 75/100/120 μm electrode separation to detect SO_2_/NO/O_2_ gases using the radio frequency voltage U_RF_ (1.5 MHz, 7W). The cathode is grounded, the radio frequency voltage U_RF_ (1.5 MHz, 7W) is applied between the extracting electrode and cathode. The potential difference between electrode generates two fields (E_1_ and E_2_) in opposing direction, which use to accelerate the position ions from ionization region to collection region and we receive the ratio of collection of positive ions in the shape of I_c1_, I_c2_ and I_c3_ as their representative sensors.


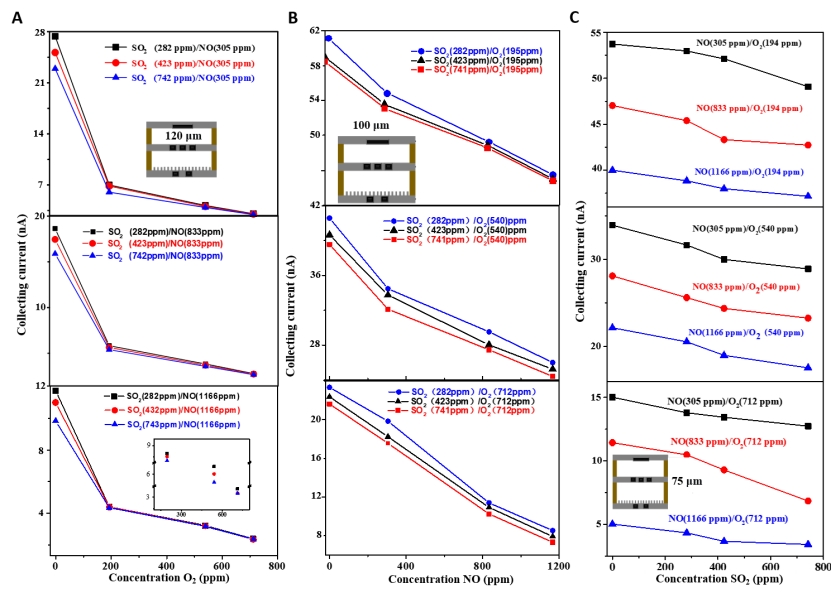


**Fig. s13**. Sensitivity characteristics of the three sensors array with the electrode spacing of 75/100/120 μm to detect SO_2_/NO/O_2_ gases mixtures in N_2_ background. **(A)** At 80 V *U*_e_, 10 V *U*_c,_ 30 ^o^C, and 22.4% RH humidity, the collecting current of the 120 μm **(B)** 100 μm and **(C)** 75 μm separation sensor decrease with the increase of the gas concentration. While the sensor with 100 μm separation shows higher sensitivity of -1.6×10^-2^ nA/ppm to 305 ppm of NO concentration, the sensor with 75 μm electrode separation shows -2.70 ×10^-3^ to 282 ppm SO_2_ concentration and the sensor with 120 μm electrode separation shows highest sensitivity of -2.2×10^-3^ to 196 ppm of O_2_ concentration.

1. Experimental results on three gases mixtures detection


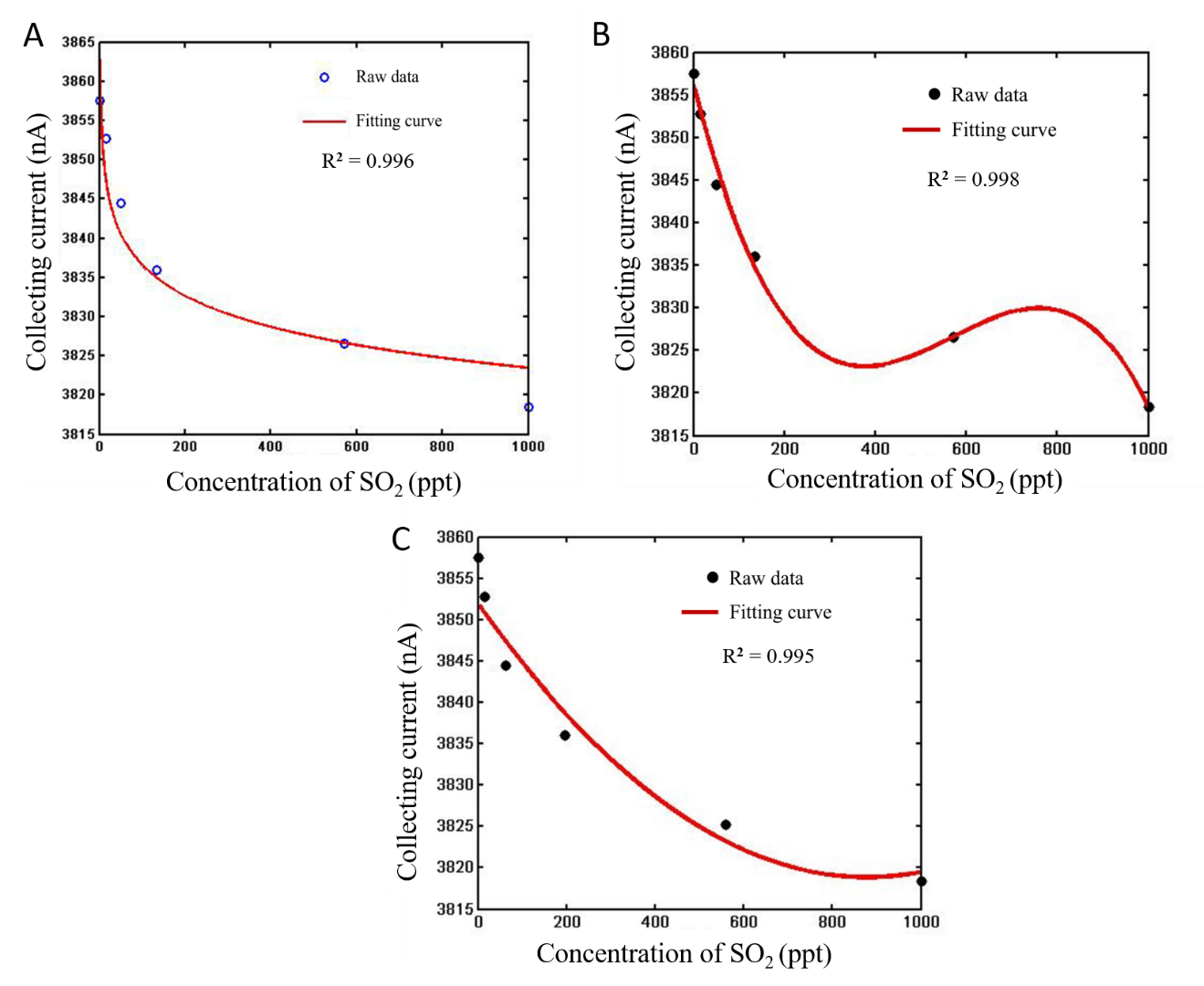


**Fig. s14**. Sensor data fitting for SO_2_ sensor. **(A)** Logarithmic function curve fitting **(B)** cubic fitting curve **(C)** Quadric fitting.

**Supplementary Reference:**

1. Sakiyama Y, Graves DB, Chang HW, et al. Plasma chemistry model of surface microdischarge in humid air and dynamics of reactive neutral species[J]. Journal of Physics D Applied Physics, 2012, 45 (42): 425201-425219(425219).
2. Brunet H, Rocca‐Serra J. Model for a Glow Discharge in Flowing Nitrogen[J]. Journal of Applied Physics, 1985, 57 (5): 1574-1581.
3. Guerra V, Sá PA, Loureiro J. Role played by the N2(A3Σu+) metastable in stationary N2 and N2-O2 discharges[J]. Journal of Physics D Applied Physics, 2001, 34 (12): 1745.
4. Zhao GB, Hu X, And MDA, et al. N Atom Radicals and N2(A3∑u+) Found To Be Responsible for Nitrogen Oxides Conversion in Nonthermal Nitrogen Plasma[J]. Indengchemres, 2004, 3 (43): 5077-5088.
5. Wang HM. Detection of sulfur dioxide by metastable N2 (A3 Sigma u+) energy transfer[J]. Proceedings of SPIE - The International Society for Optical Engineering, 2005, 5832.
6. Zhao G., Hu X., Argyle M. D., et al. Effect of CO2 on nonthermal-plasma reactions of nitrogen oxides in N2. 1. PPM-level concentrations[J]. Industrial & engineering chemistry research,2005,44(11):3925-3934.
7. Tatarova E, Dias F M, Gordiets B et al. Molecular dissociation in N2–H2 microwave discharges[J]. Plasma Sources Science and Technology, 2005, 14 (1): 19.
8. Stoykov S, Eggs C, Kortshagen U. Plasma chemistry and growth of nanosized particles in a C2H2 RF discharge[J]. Journal of Physics D: Applied Physics, 2001, 34 (14): 2160.
9. Mao M, Benedikt J, Consoli A et al. new pathways for nanoparticle formation in acetylene dusty plasmas: a modelling investigation and comparison with experiments[J]. Journal of Physics D: Applied Physics, 2008, 41 (22): 225201.
